# Supplementary material for: SRSF1 promotes vascular smooth muscle cell proliferation through a Δ133p53/EGR1/KLF5 pathway
Source: Nat Commun. 2017 Aug 11;8:16016. doi: 10.1038/ncomms16016 (PMC5561544; doi:10.1038/ncomms16016)
Supplement: Supplementary Information [file ncomms16016-s1.pdf]

Type of file: PDF
Title of file for HTML: Supplementary Information
Description: Supplementary Figures and Supplementary Tables.

Type of file: PDF
Title of file for HTML: Peer Review File
Description:

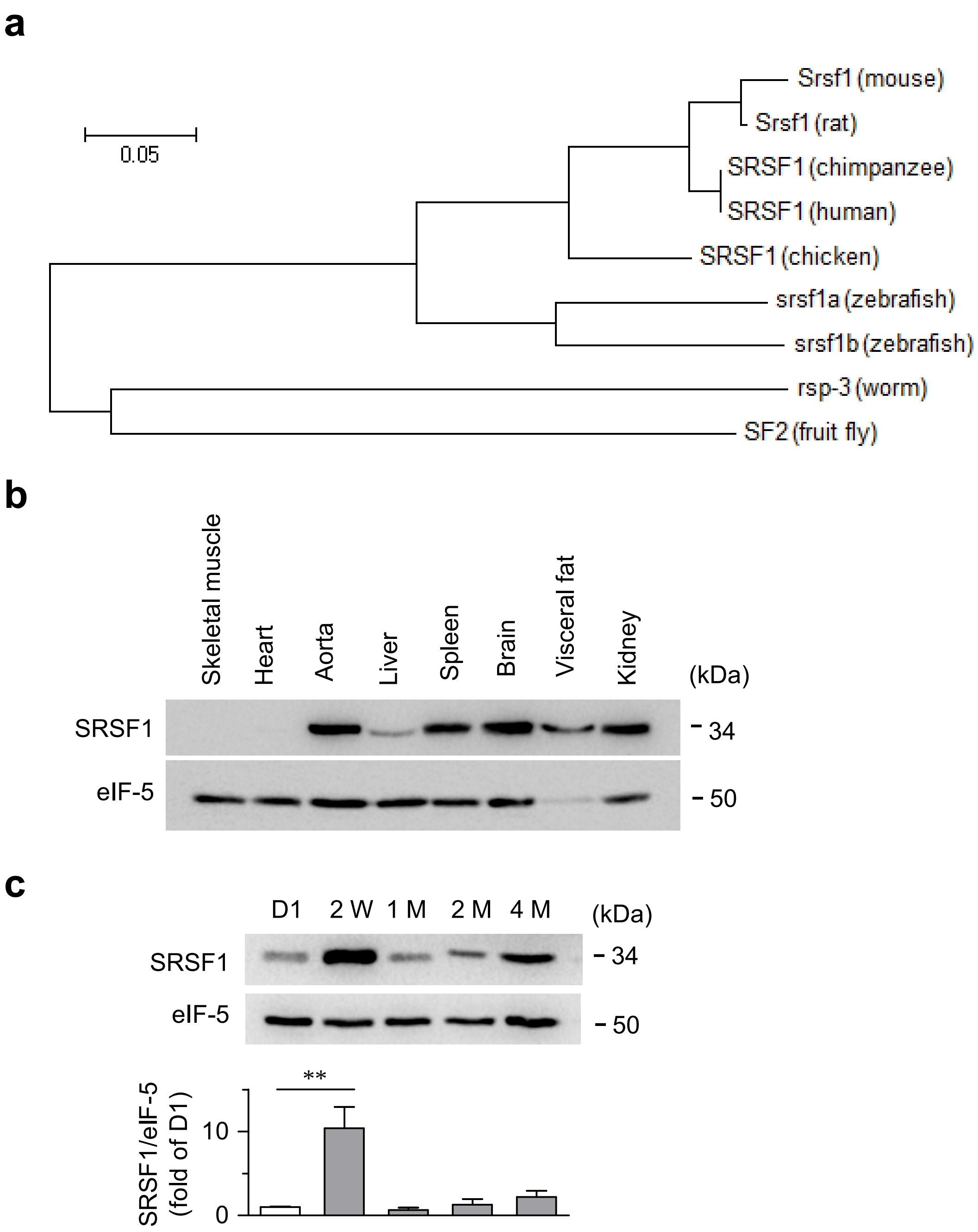


**Supplementary Figure 1 | Expression profile of Srsf1 in aorta.** (**a**) Molecular phylogenetic tree of SRSF1 in selected model organisms. The branch length shows the distance as measured by the average nucleotide substitutions per site. (**b**) Representative western blots showing that SRSF1 is expressed in the aorta in 12-14 week-old rats. (**c**) Western blots and analysis of aortic SRSF1 expression at birth (D1), two weeks (2 W), one month (1 M), two months (2 M), and four months (4 M) after birth; *n* = 5 per group. eIF-5 served as internal control. ***P* <0.01, one-way ANOVA (**c**). Data are mean ± s.e.m. of four (**c**) independent experiments.

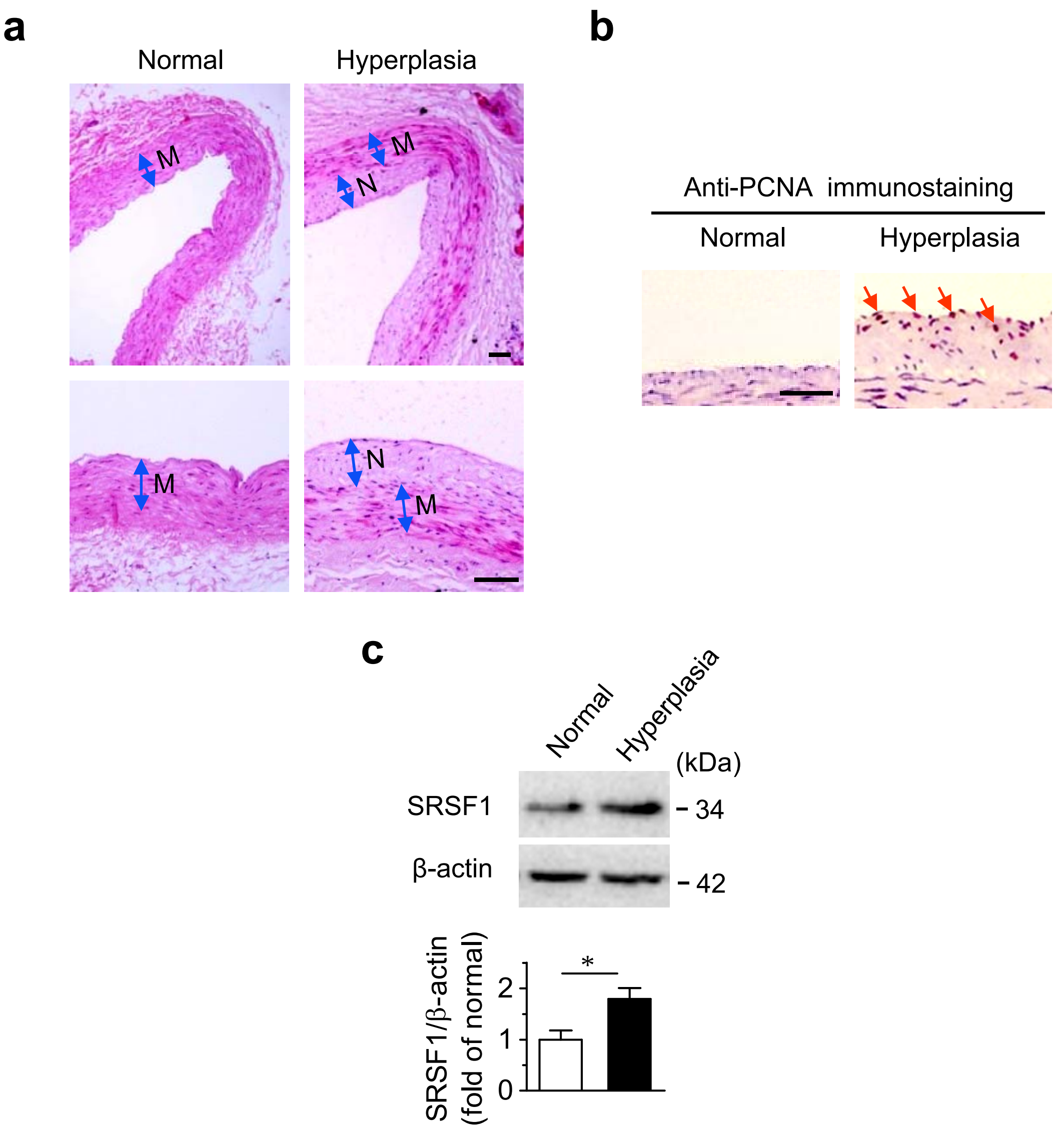


**Supplementary Figure 2 | Increased SRSF1 protein in hyperplasia human artery.** (**a**) Representative photomicrographs of hematoxylin/eosin staining of the neointima area of human internal mammary arteries (N, neointima; M, media) collected during cardiac bypass surgery (×100; scale bars, 50 μm). (**b**) Representative photomicrographs of immunohistochemical staining showing PCNA-positive cells in a human artery with hyperplasia (scale bar, 25 μm). Arrows indicate PCNA-positive cells (dark brown). (**c**) Representative western blots and averaged data showing the SRSF1 levels in human arteries; $n$ = 5 per group. *$P$<0.05, Student's $t$-test (**c**). Data are mean ± s.e.m. of three independent experiments (**c**).

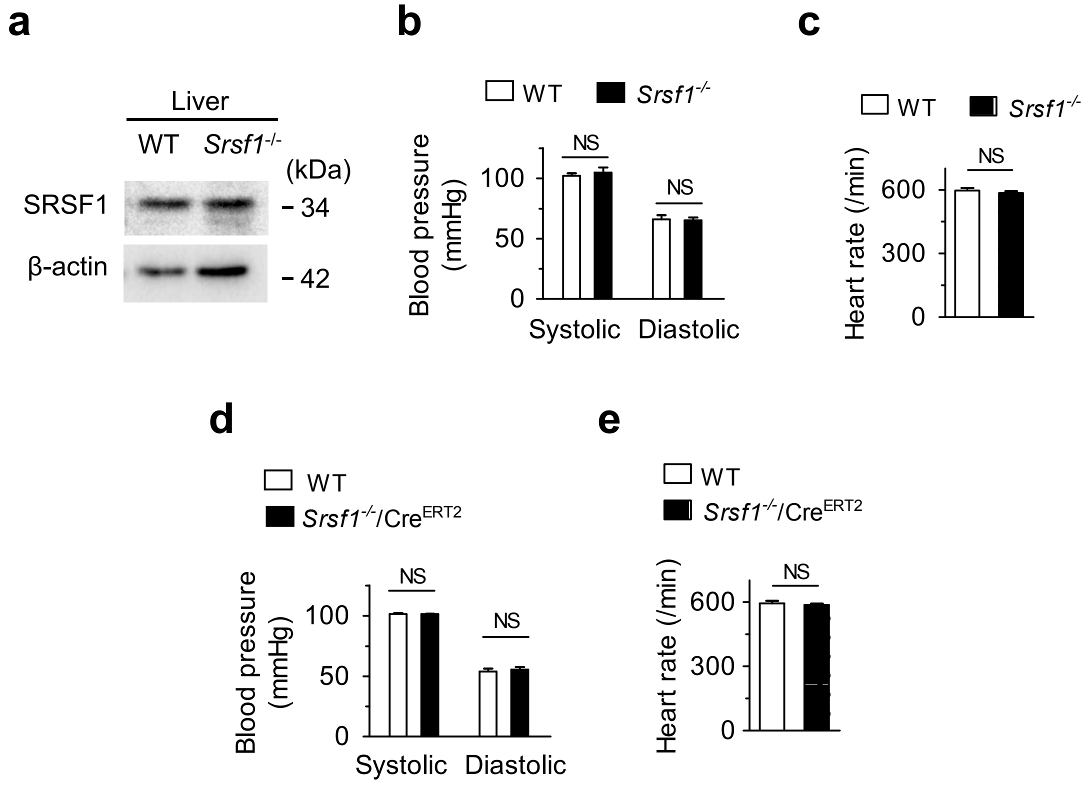


**Supplementary Figure 3 | Genotyping and blood pressure of smooth muscle cell-specific *Srsf1* knockout (*Srsf1*$^{-/-}$) mice and inducible smooth muscle cell-specific *Srsf1* knockout (*Srsf1*$^{-/-}$/Cre$^{ERT2}$) mice.** (**a**) Representative western blots demonstrating the unaltered expression of SRSF1 at the protein level in liver of WT and *Srsf1*$^{-/-}$ mice. (**b**,**c**) Systolic and diastolic blood pressure (**b**), and heart rate (**c**) of WT and *Srsf1*$^{-/-}$mice at 10 weeks of age; *n* = 10 per group. (**d**,**e**) Systolic and diastolic blood pressure (**d**), and heart rate (**e**) of WT and *Srsf1*$^{-/-}$/Cre$^{ERT2}$ at 10 weeks of age; *n* = 12 per group. NS, not significant, Student's *t*-test (**b-e**).

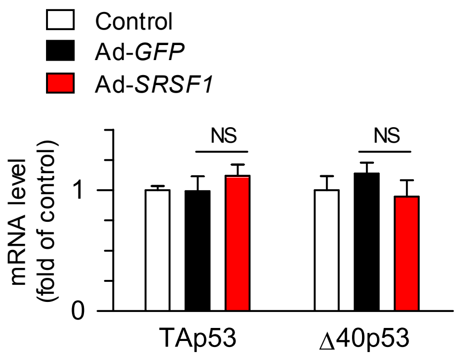


**Supplementary Figure 4 │ SRSF1 overexpression did not affect the expression of full-length p53 and Δ40p53.** There are at least 3 subclasses of human p53 isoforms due to N-terminal truncation, TAp53 (N-terminal with completely conserved transactive domain), Δ40p53 (truncated N-terminal missing the first 39 amino-acids), and Δ133p53 (truncated N-terminal missing the first 132 amino-acids). 'Δ40p53' primers qualify the Δ40p53 isoform subclass; 'TAp53' primers qualify the Δ40p53 isoform subclass and the TAp53 isoform subclass (mainly includes full-length p53). Real-time PCR showing the mRNA levels of the TAp53 and Δ40p53 isoforms in HASMCs infected with Ad-*GFP* or Ad-*SRSF1* (m.o.i. 100, 48 h); *n* = 8 per group. NS, not significant, one-way ANOVA. Data are mean ± s.e.m. of four independent experiments.

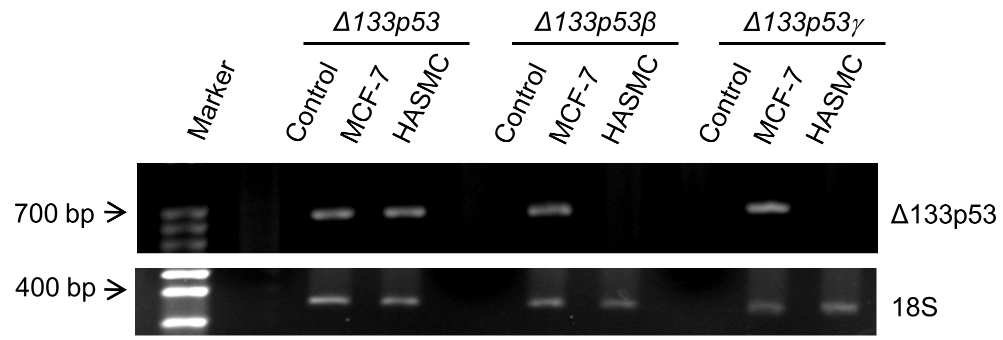


**Supplementary Figure 5│ Amplification of Δ133p53 isoform mRNAs by nested PCR in human cells.** MCF-7, human breast adenocarcinoma cell line, positive control; HASMC, human aortic smooth muscle cell; Con, negative control, dd$H_2O$ was used instead of cDNA. Specific primers were used for amplifying *Δ133p53* (*Δ133p53α*), *Δ133p53β* or *Δ133p53γ* by nested RT-PCR (see Supplementary Tables 6 and 7). PCR products (20 μL) were migrated on 1% agarose gel. Marker, 100 bp DNA ladder. DNA sequencing of the bands obtained was performed to verify the isoforms. While all three *Δ133p53* isoforms (α, β and γ) present in MCF-7 cells, only Δ133p53α was detectable in HASMCs.

```
Mouse Δ157p53  - - - - - - - - - - - - - - - - - - - - - MAIYKKSQHMTEVVRRCPHHERCSDGDGLAPPQ  33
Human Δ133p53  MFCQLAKTCPVQLWVDSTPPPGTRVRAMAIYKQSQHMTEVVRRCPHHERCSDSDGLAPPQ  60

               HLIRVEGNLYPEYLEDRQTFRHSVVVPYEPPEAGSEYTTIHYKYMCNSSCMGGMNRRPIL  93
               HLIRVEGNLRVEYLDDRNTFRHSVVVPYEPPEVGSDCTTIHYNYMCNSSCMGGMNRRPIL  120

               TIITLEDSSGNLLGRDSFEVRVCACPGRDRRTEEENFRKKEVLCPELPPGSAKRALPTCT  153
               TIITLEDSSGNLLGRNSFEVRVCACPGRDRRTEEENLRKKGEPHHELPPGSTKRALPNNT  180

               SASPPQKKKPLDGEYFTLKIRGRKRFEMFRELNEALELKDAHATEESGDSRAHSSYLKTK  213
               SSSPQPKKKPLDGEYFTLQIRGRERFEMFRELNEALELKDAQAGKEPGGSRAHSSHLKSK  240

               KGQSTSRHKKTMVKKVGPDSD  234
               KGQSTSRHKKLMFKTEGPDSD  261
```

**Supplementary Figure 6** | Protein alignment between human Δ133p53 and mouse Δ157p53 (named MΔ133p53 in this study) illustrating the high degree of homology between the two protein sequences.

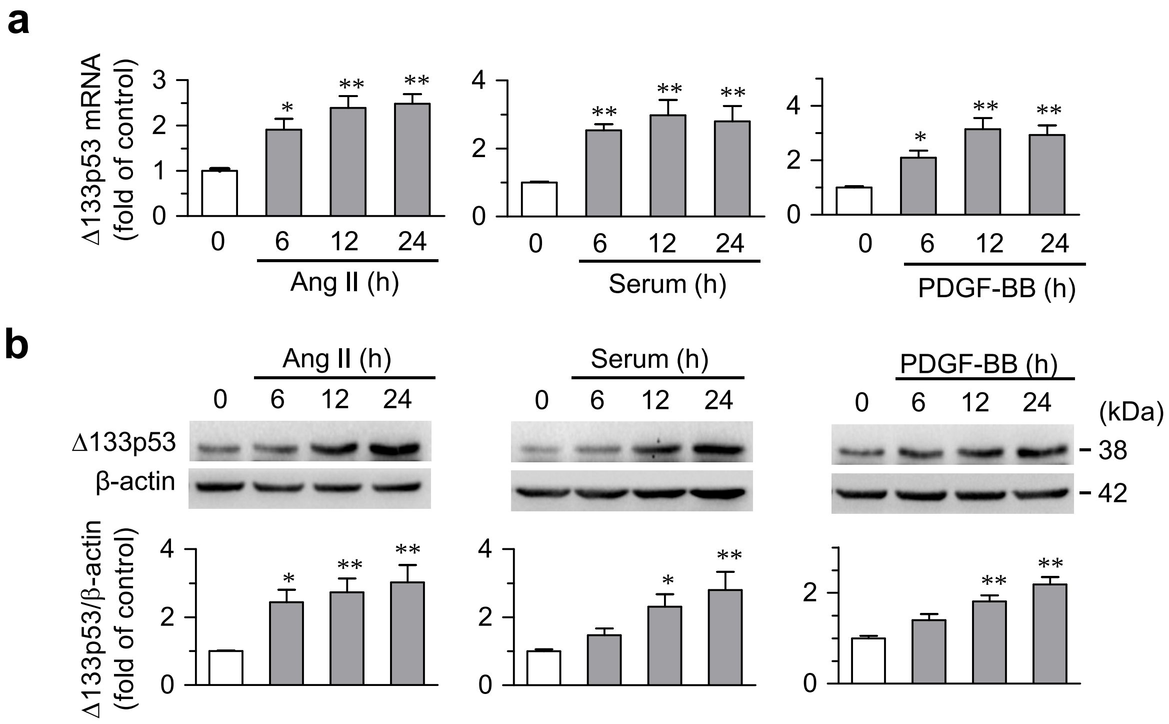


**Supplementary Figure 7 | Δ133p53 expression was increased in proliferating smooth muscle cells.** (**a**) Real-time PCR data showing the mRNA levels of Δ133p53 in HASMCs treated with Ang II (200 nM), serum (10% FBS), or PDGF-BB (10 μg/L) at 6, 12, and 24 h; *n* = 8 per group. (**b**) Representative western blots and averaged data showing the Δ133p53 levels in HASMCs treated as in (**a**); *n* = 7 per group. *$P$ <0.05, **$P$ <0.01 *vs* control group, one-way ANOVA. Data are mean ± s.e.m. of five independent experiments.

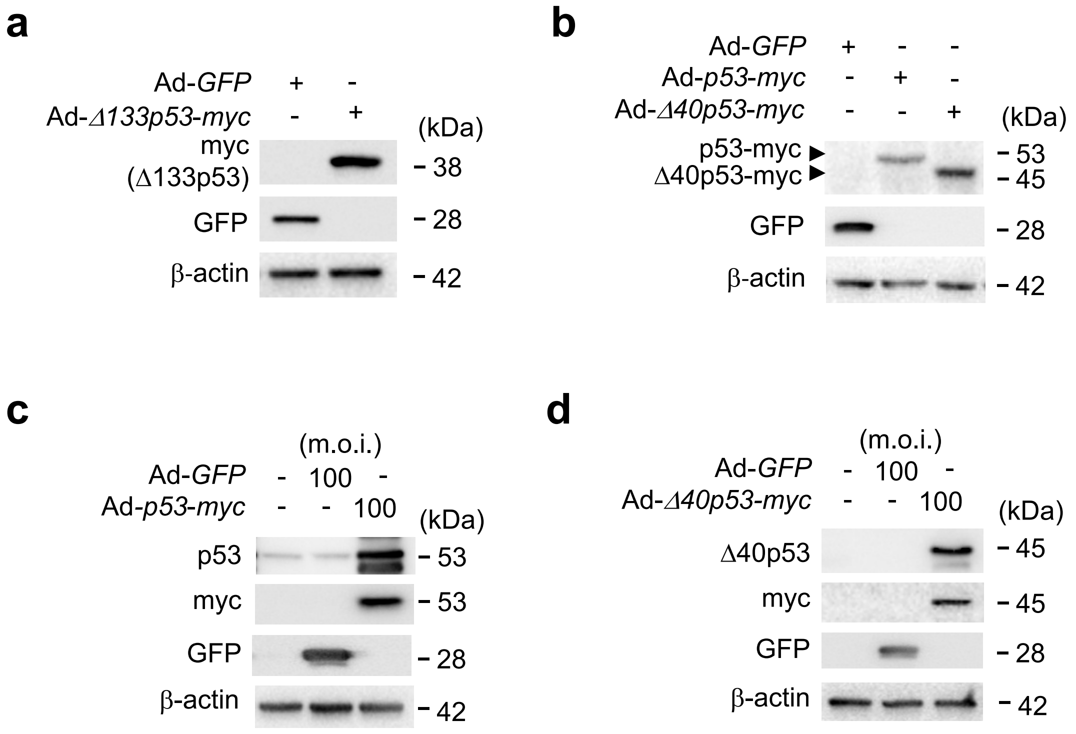


**Supplementary Figure 8** │ Representative western blots showing expression of indicated genes in rat carotid arteries at 4 days after Ad-*Δ133p53-myc* (**a**), Ad-*p53-myc* or Ad-*Δ40p53-myc* (**b**) delivery (*n* = 5 per group). (**c**,**d**) Representative western blots showing the p53 (**c**) or Δ40p53 (**d**) expression in cultured HASMCs infected with Ad-*GFP*, Ad-*p53*, or Ad-*Δ40p53* for 48 h (m.o.i. 100, *n* = 5 per group).

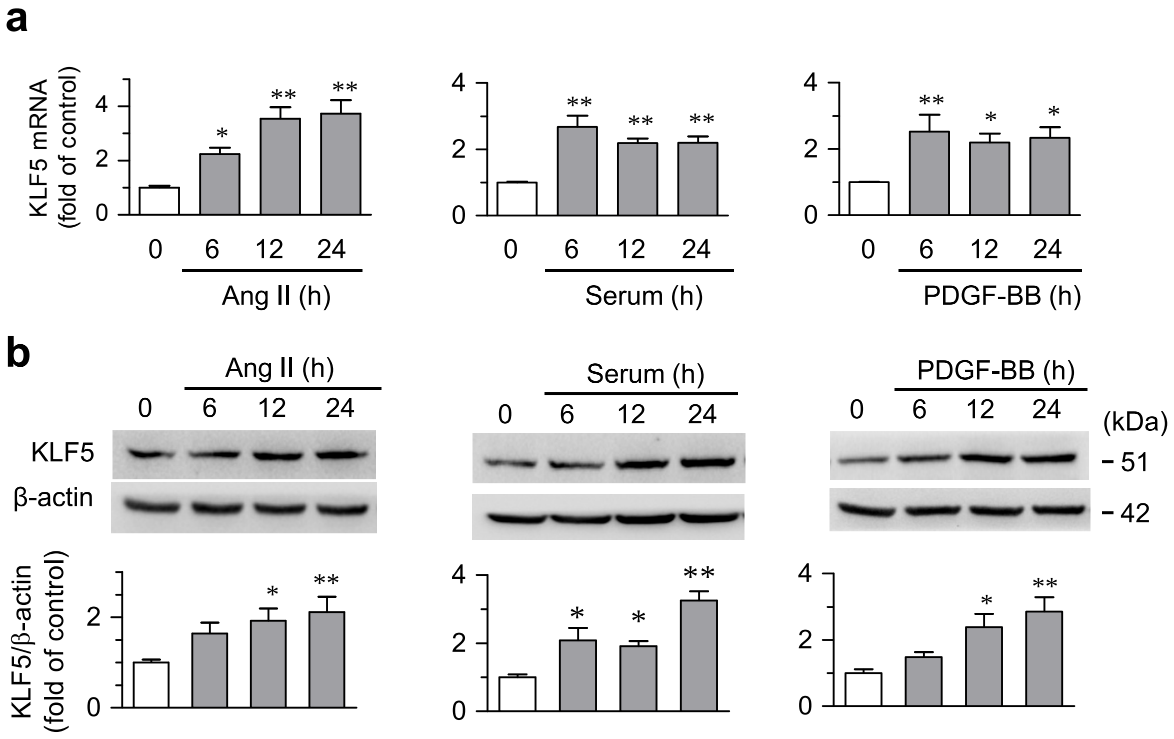


**Supplementary Figure 9 | KLF5 expression was increased in proliferating smooth muscle cells.** (**a**) Real-time PCR data showing the mRNA levels of KLF5 in HASMCs treated with Ang II (200 nM), serum (10% FBS), or PDGF-BB (10 μg $L^{-1}$) at 6, 12, and 24 h; *n* = 8 per group. (**b**) Representative western blots and averaged data showing KLF5 levels in HASMCs treated as in (**a**); *n* = 7 per group. **P* <0.05, ***P* <0.01 *vs* control group, one-way ANOVA. Data are mean ± s.e.m. of four independent experiments.

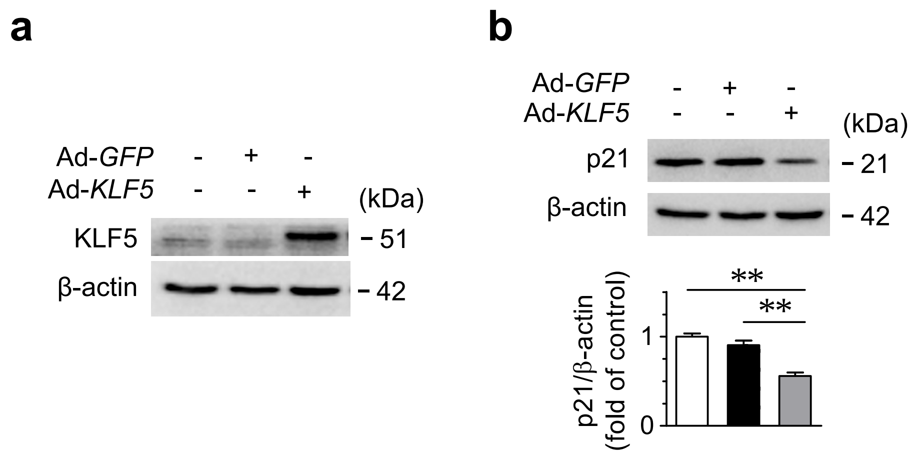


**Supplementary Figure 10 │ KLF5 decreased p21 expression.** (**a**) Representative western blots showing the KLF5 expression in HASMCs infected with Ad-*GFP* or Ad-*KLF5* (m.o.i. 100, 48 h) ($n$ = 5 per group). (**b**) Representative western blots and averaged data showing p21 levels in HASMCs treated as in (**a**); $n$ = 5 per group. $^{**}P$ <0.01, one-way ANOVA (**b**). Data are mean ± s.e.m. of four independent experiments.

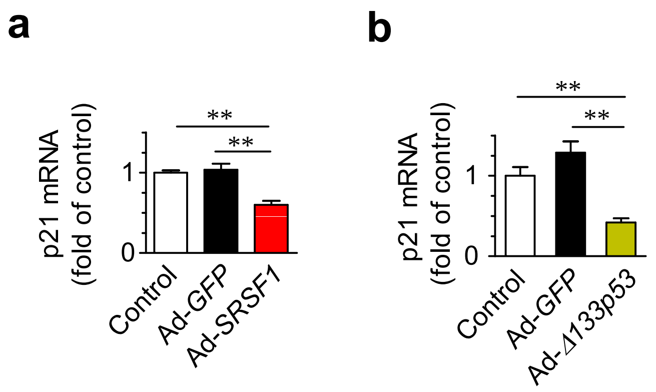


**Supplementary Figure 11 │ Overexpression of SRSF1 or Δ133p53 decreased p21 expression.** (**a**) Real-time PCR data showing the mRNA levels of p21 in HASMCs infected with Ad-*SRSF1*; *n* = 7 per group. (**b**) Real-time PCR data showing the mRNA levels of p21 in HASMCs infected with Ad-*Δ133p53*; *n* = 12 per group. ***P* <0.01, one-way ANOVA. Data are mean ± s.e.m. of five independent experiments.

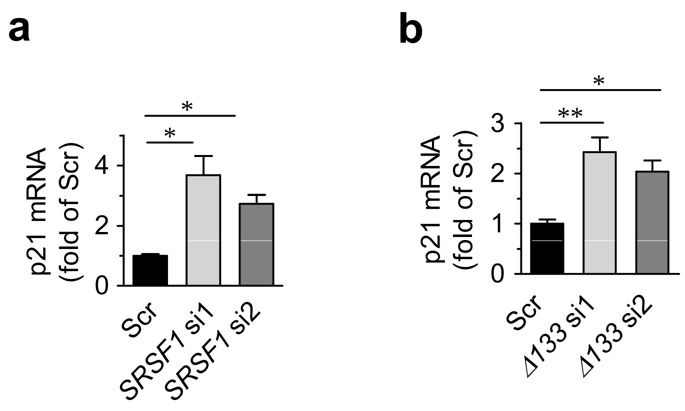


**Supplementary Figure 12 │ Knockdown of SRSF1 or Δ133p53 increased p21 expression.** (**a**) Real-time PCR data showing the mRNA levels of p21 in HASMCs infected with scrambled or *SRSF1* siRNAs (*SRSF1* si1 and *SRSF1* si2); *n* = 8 per group. (**b**) Real-time PCR data showing the mRNA levels of p21 in HASMCs infected with scrambled or *Δ133p53* siRNAs (*Δ133* si1 and *Δ133* si2); *n* = 7 per group. Scr indicates scrambled siRNA control. *$P$ <0.05, **$P$ <0.01, one-way ANOVA. Data are mean ± s.e.m. of five independent experiments.

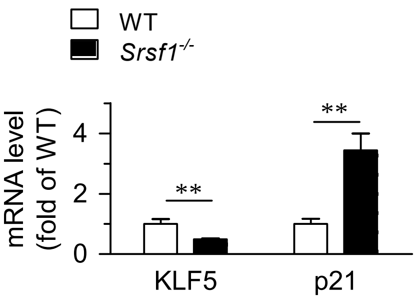


**Supplementary Figure 13** | Real-time PCR data showing the mRNA levels of KLF5 and p21 in arteries from WT and *Srsf1*$^{-/-}$ mice; *n* = 8 per group. ***P* <0.01, Student's *t*-test. Data are mean ± s.e.m. of three independent experiments.

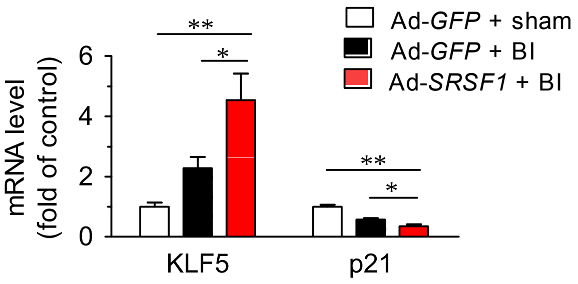


**Supplementary Figure 14** | Real-time PCR data showing the mRNA levels of KLF5 and p21 in rat carotid arteries transfected with Ad-*GFP* or Ad-*SRSF1*, one week after balloon injury; *n* = 8/group. **P* <0.05, ***P* <0.01, one-way ANOVA. Data are mean ± s.e.m. of five independent experiments.

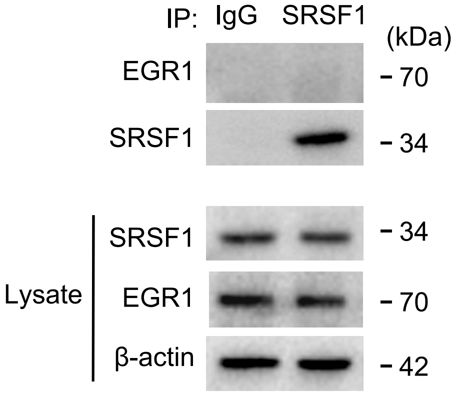


**Supplementary Figure 15** │ Representative western blots of co-immunoprecipitation between SRSF1 and EGR1 in HASMCs (*n* = 5).

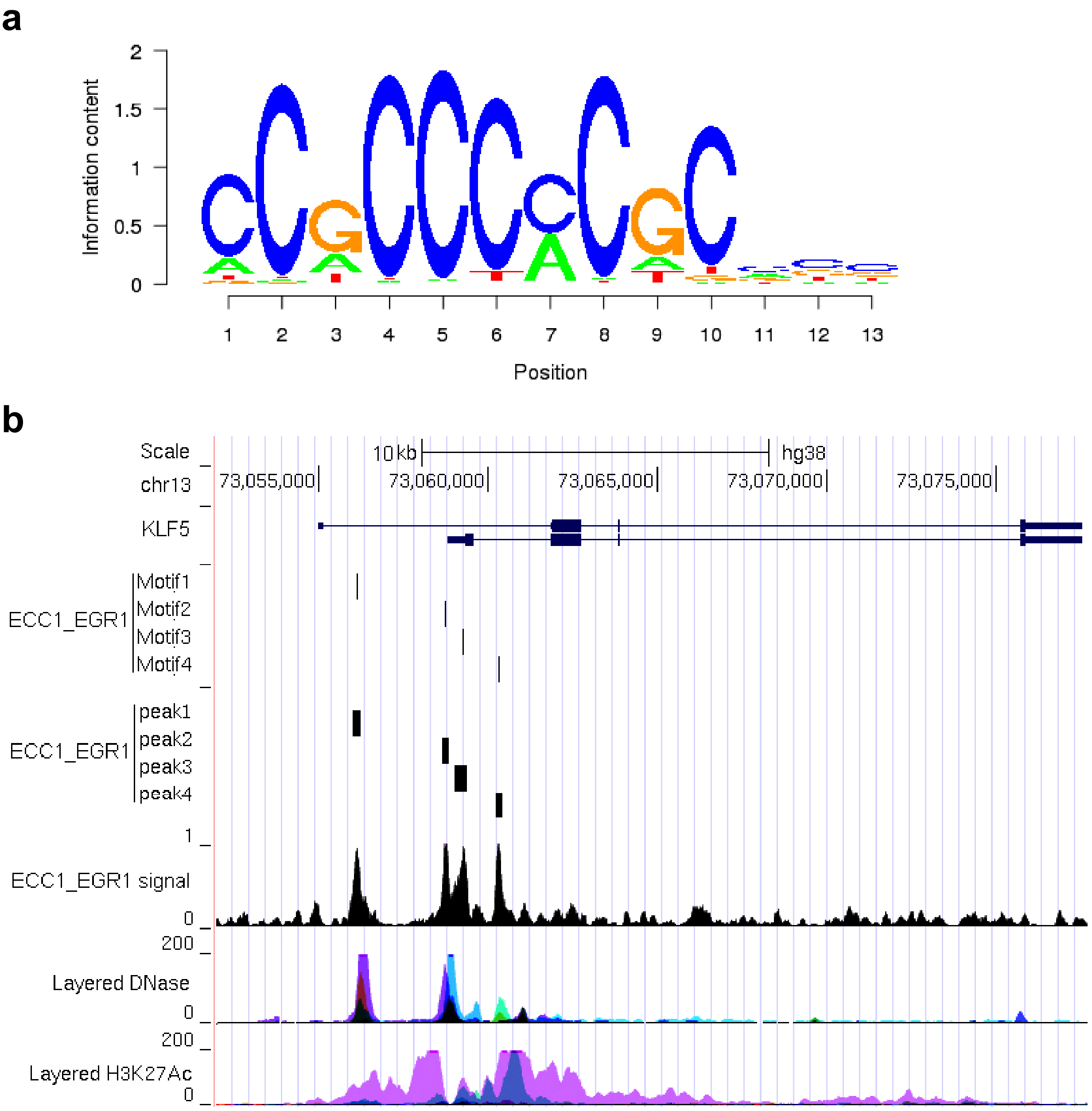


**Supplementary Figure 16 | Annotation of the predicted EGR1 binding sites in the KLF5 gene using the Cistrome data browser.** (**a**) Sequence logo of the EGR1 binding motif visualized using Cistrome. (**b**) The KLF5 upstream genomic region (chr13:73054976-73077542:GRCh38/hg38) is illustrated using the UCSC genome browser. ChIP-seq signals and peaks of H3K27ac, and DNAse-seq peaks were extracted from the Cistrome data. The read density of ChIP-seq signals is indicated in the Y-axis. ChIP-seq peaks of EGR1 in human cell line ECC1 from the Cistrome data and 4 predicted EGR1 binding motifs are indicated.

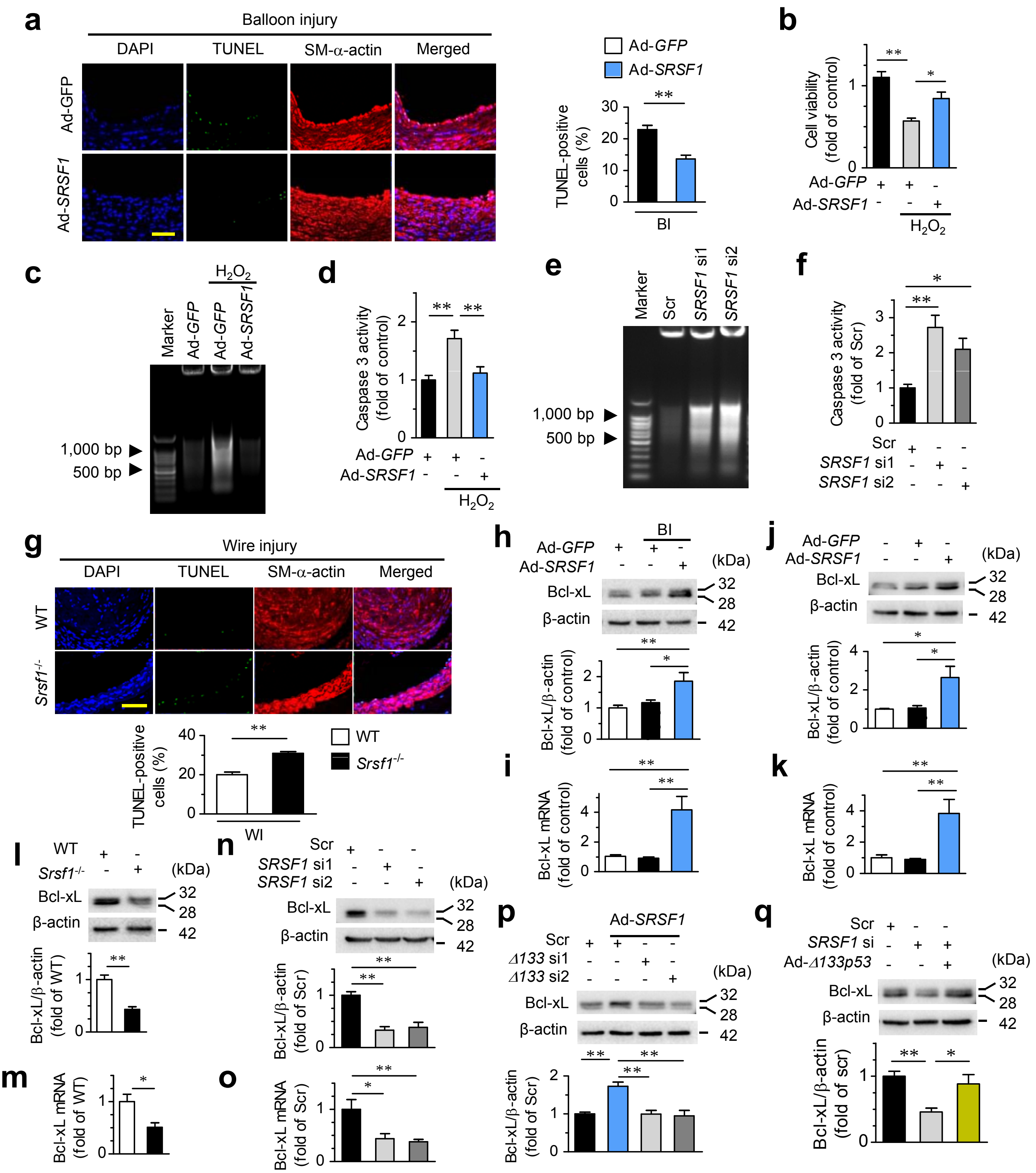


**Supplementary Figure 17│ SRSF1 blockade of apoptosis contributes to neointima formation induced by wire-injury.** (**a**) Representative photomicrographs (**left**) and averaged data (**right**) for TUNEL staining of rat carotid arteries transfected with Ad-*GFP* or Ad-SRSF1, two weeks after balloon injury; Green, TUNEL-positive VSMC nuclei; blue, DAPI-stained nuclei; red, VSMCs labeled with antibody to SM-α-actin; scale bar, 50 μm. *n* = 9 per group. (**b**,**c**,**d**) Cell viability (**b**), DNA laddering (**c**), and caspase 3 activity (**d**) in HASMCs treated with $H_2O_2$ (400 μM, 6 h) with or without Ad-*SRSF1* overexpression; *n* = 5 (**c**) or 9 samples (**b**,**d**). (**e**,**f**) DNA laddering (**e**) and caspase 3

activity (**f**) in HASMCs infected with *SRSF1* siRNAs; *n* = 5 (**e**) or 9 samples (**f**). (**g**) Representative photomicrographs and averaged data for TUNEL staining of carotid artery sections from WT and *Srsf1*$^{-/-}$ mice subjected to wire injury (4 weeks); Green, TUNEL-positive VSMC nuclei; blue, DAPI-stained nuclei; red, VSMCs labeled with antibody to SM-α-actinin; scale bar, 50 μm; *n* = 9 per group. (**h**) Representative western blots and averaged data showing Bcl-xL levels in rat carotid arteries transfected with Ad-*GFP* or Ad-*SRSF1* 4 days after balloon injury; *n* = 6 per group. (**i**) Real-time PCR data showing the Bcl-xL mRNA in rat carotid arteries transfected with Ad-*GFP* or Ad-*SRSF1*, one week after balloon injury; *n* = 8 per group. (**j**) Representative western blots and averaged data showing Bcl-xL levels in HASMCs with SRSF1 overexpression; *n* = 6 per group. (**k**) Real-time PCR data showing the Bcl-xL levels in HASMCs infected with Ad-*GFP* or Ad-*SRSF1*; *n* = 8 per group. (**l**) Representative western blots and averaged data showing Bcl-xL levels in arteries from *Srsf1*$^{-/-}$ and WT control mice; *n* = 7 per group. (**m**) Real-time PCR data showing the Bcl-xL levels in aortas from WT and *Srsf1*$^{-/-}$ mice; *n* = 8 per group. (**n**) Representative western blots and averaged data showing KLF5 levels in HASMCs with SRSF1-knockdown by *SRSF1* siRNAs; *n* = 5 per group. (**o**) Real-time PCR data showing the Bcl-xL levels in HASMCs infected with scrambled or *SRSF1* siRNAs; *n* = 8 per group. (**p**) Representative western blots and averaged data showing the Bcl-xL levels in HASMCs infected with scrambled or *Δ133p53* siRNAs (*Δ133* si1 and *Δ133* si2) with or without of Ad-*SRSF1* overexpression; *n* = 9 per group. (**q**) Representative western blots and averaged data showing the Bcl-xL levels in HASMCs infected with scrambled or *SRSF1* siRNA1 (*SRSF1* si) with or without overexpression of Ad-*Δ133p53*; *n* = 7 per group. Scr indicates scrambled siRNA control. BI, balloon injury. WI, wire injury. **P* <0.05, ***P* <0.01, Student's *t*-test (**a**,**g**,**l**,**m**) or one-way ANOVA (**b**,**d**,**f**,**h-k**,**n-q**). Data are mean ± s.e.m. of three (**m**) or four (**a-g**,**o**) or five (**h-l**,**n-q**) independent experiments.

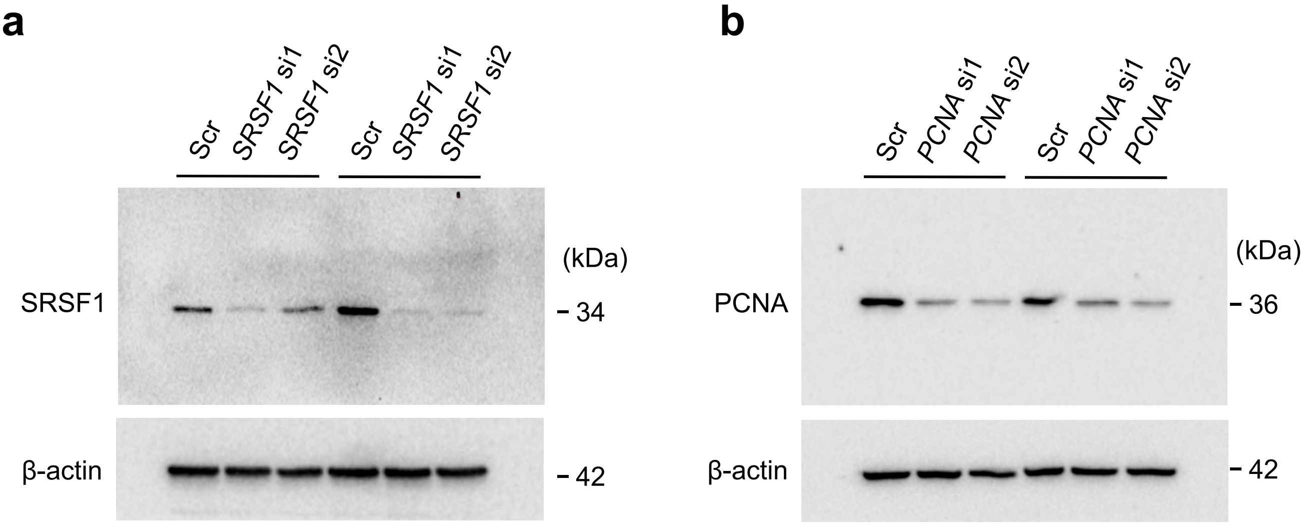


**Supplementary Figure 18 │ Knock down of SRSF1 or PCNA in HASMCs.** (**a**) Representative western blots showing SRSF1 levels in HASMCs with SRSF1-knockdown by *SRSF1* siRNAs. Antibody for SRSF1 from Invitrogen (catalog number 32-4500), recognizes the first 97 amino acids of SRSF1. (**b**) Representative western blots showing PCNA levels in HASMCs with PCNA-knockdown by *PCNA* siRNAs. Antibody for PCNA from Signaling Technology (catalog number 2586), recognizes the protein region within the amino acid residues 111-125.

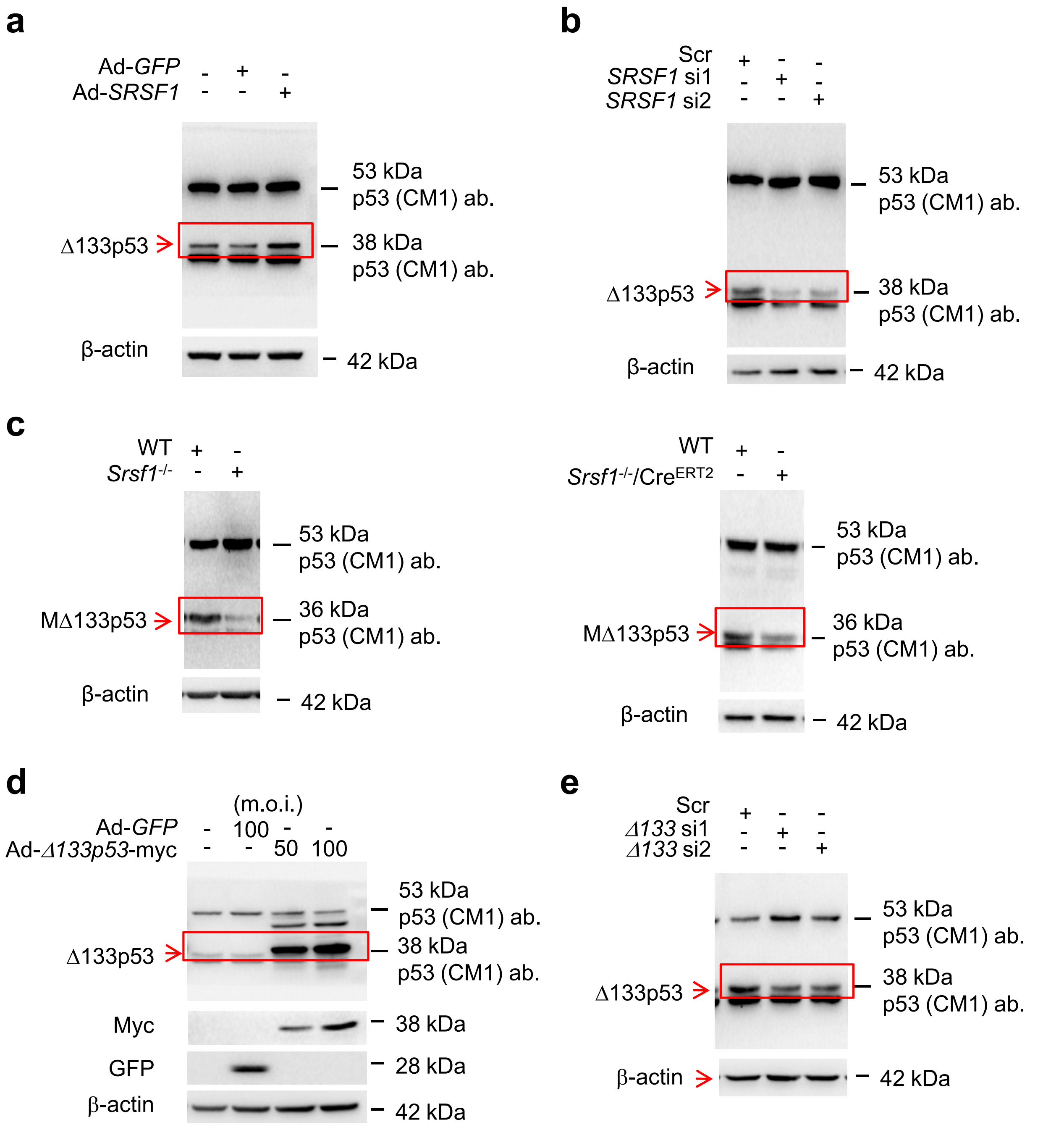


**Supplementary Figure 19 | Representative full-length gels showing all p53 isoforms.** (**a**,**b**) Gels for p53 isoforms in HASMCs with SRSF1-overexpression (**a**) and SRSF1-knockdown by *SRSF1* siRNAs (*SRSF1* si1 and *SRSF1* si2) (**b**). (**c**) Gels for p53 isoforms in vessels from *Srsf1*$^{-/-}$ and WT control mice at 56 days after birth. (**d**,**e**) Gels for p53 isoforms in HASMCs with Δ133p53-overexpression (**d**) or Δ133p53-knockdown by *Δ133p53* siRNAs (*Δ133* si1 and *Δ133* si2) (**e**). The antibody we used to detect Δ133p53 (and Δ157p53) was p53 (CM1) antibody (Vector Laboratories, VP-P955), a rabbit polyclonal antibody raised against recombinant full-length human p53 protein that recognizes all p53 isoforms.

**Supplementary Figure 20│ Uncropped images of blots in Figure 1-9 and Supplementary Figure 1-19.**

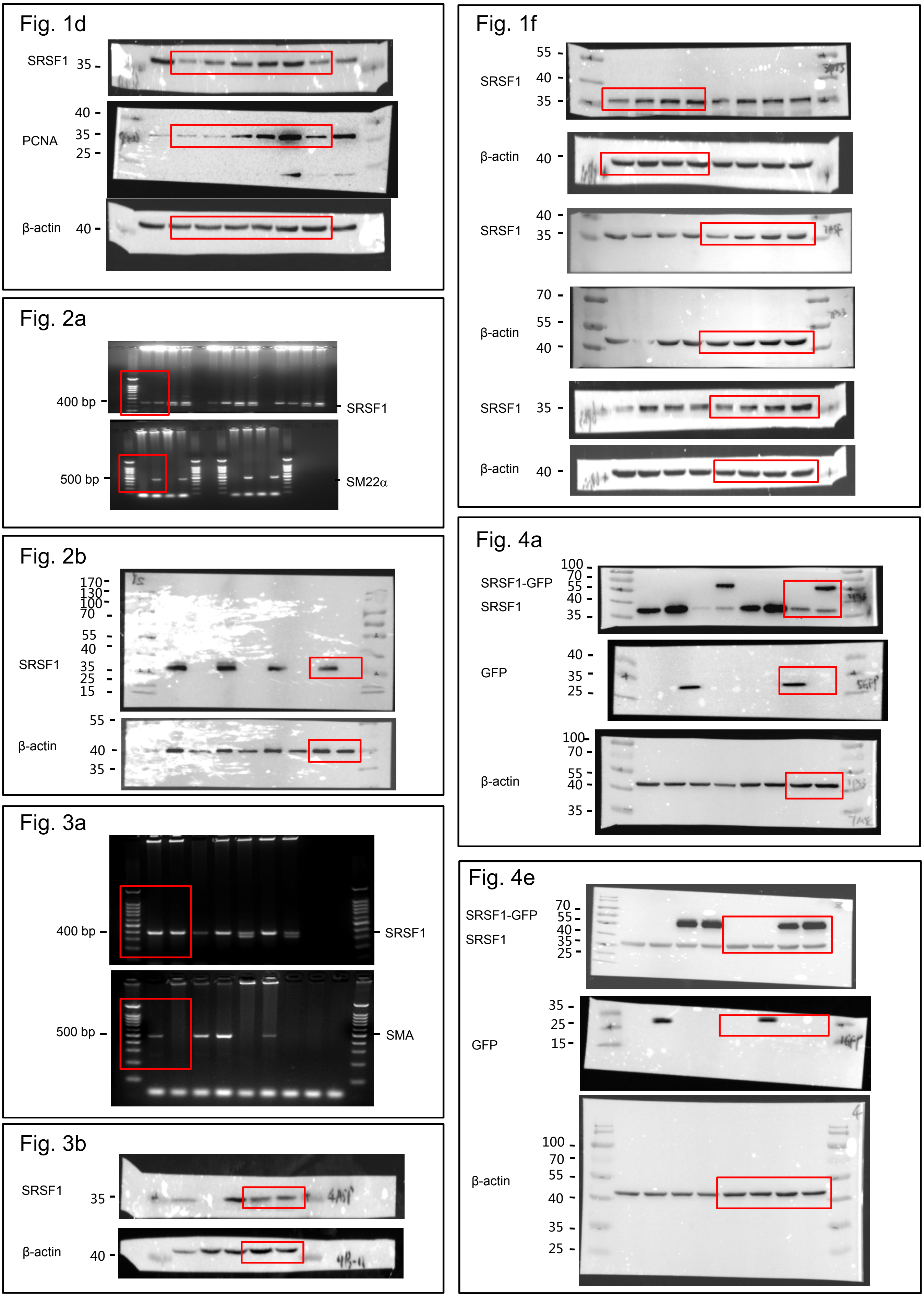

Fig. 4j

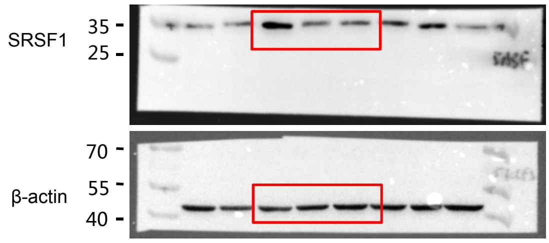


Fig. 5e

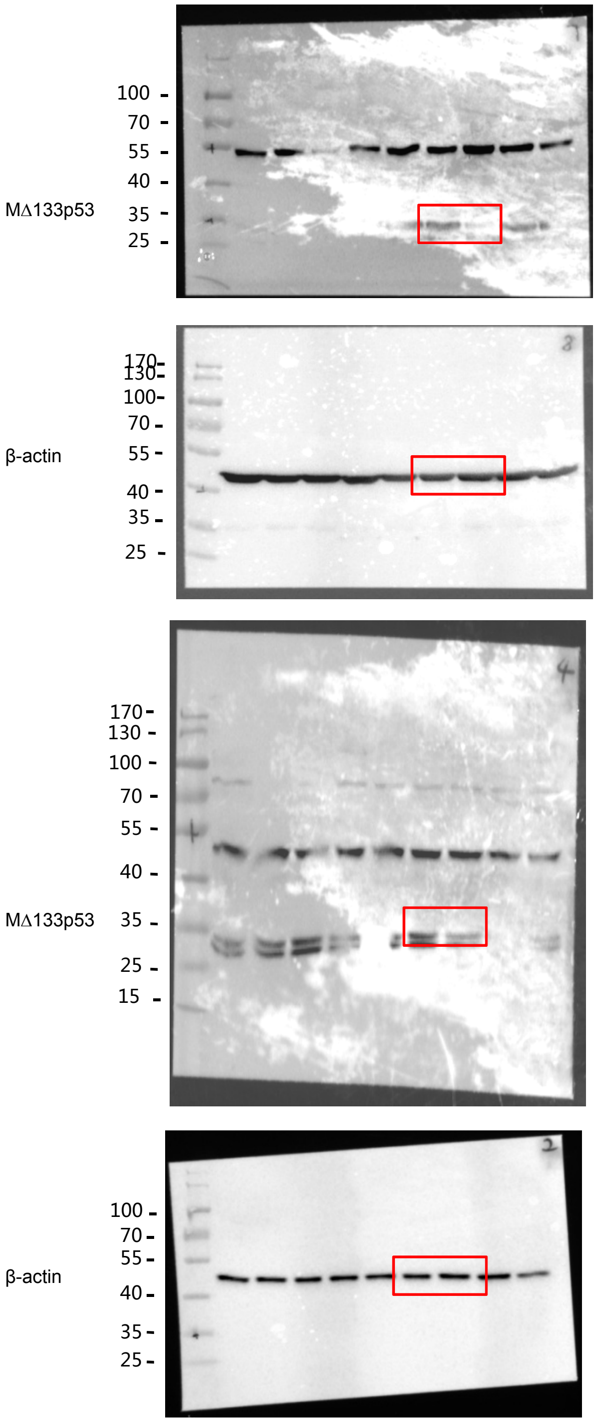


Fig. 4f

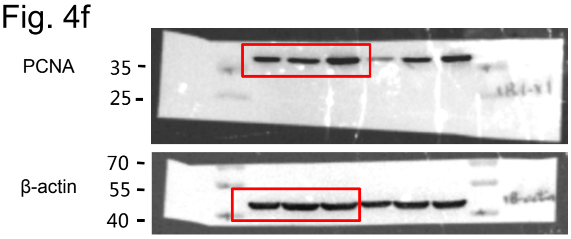


Fig. 4k

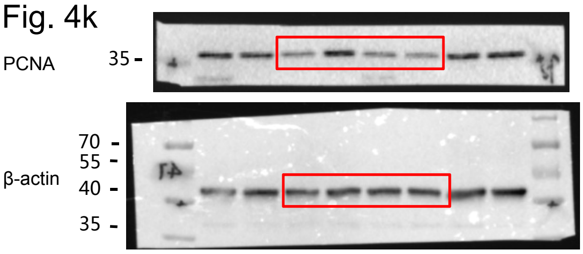


Fig. 5b

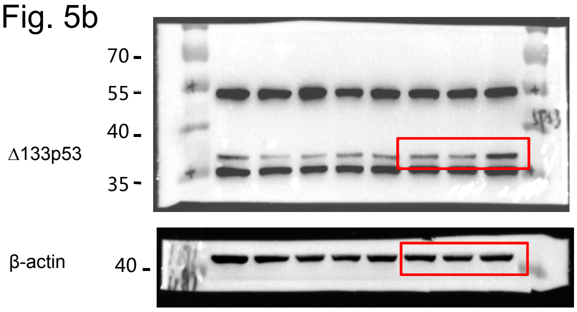


Fig. 5d

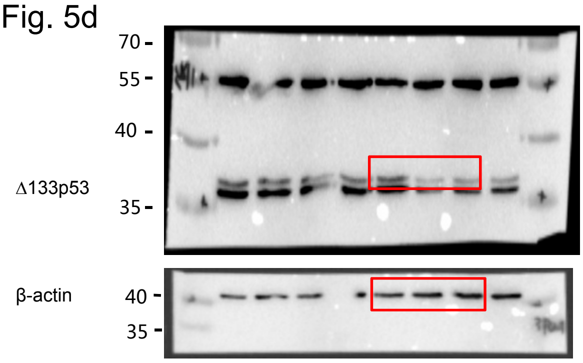


Fig. 5f

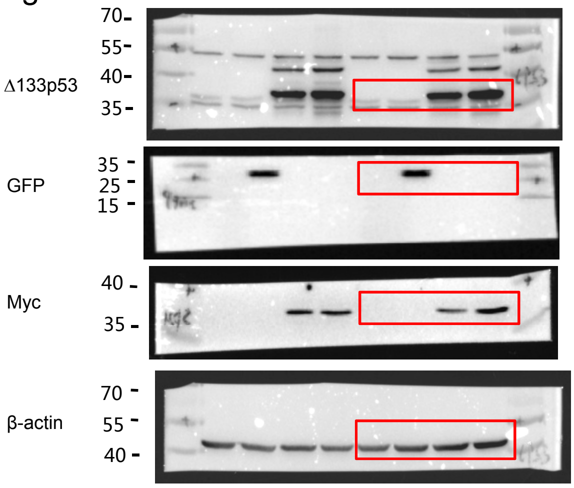

Fig. 5g

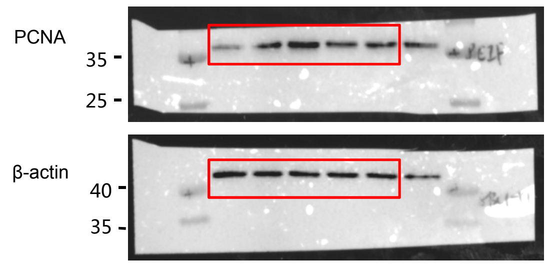


Fig. 5n

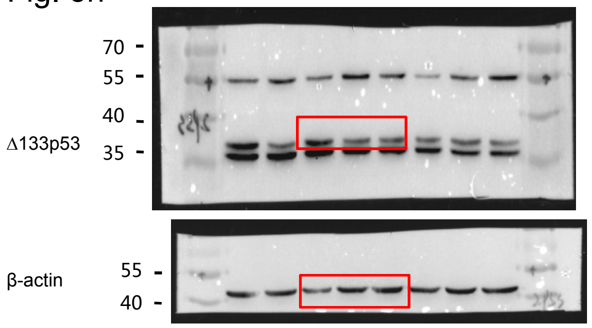


Fig. 5o

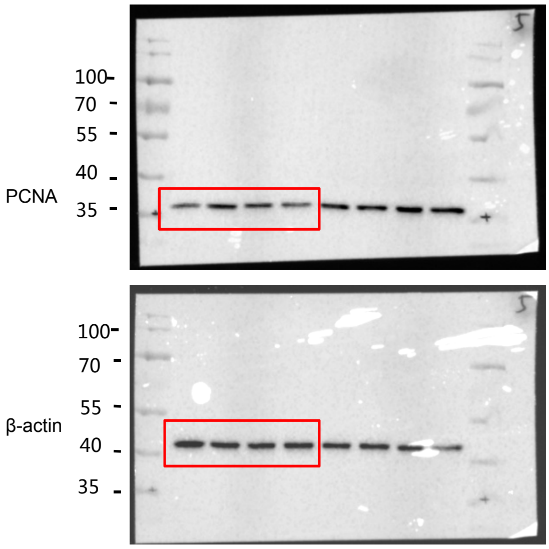


Fig. 5r

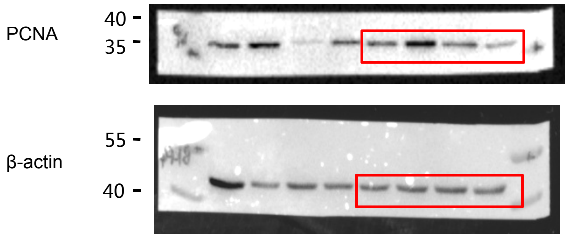


Fig. 6e

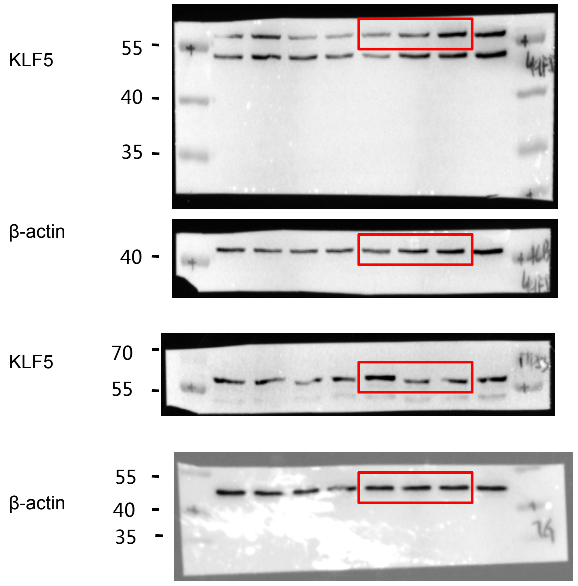


Fig. 6c

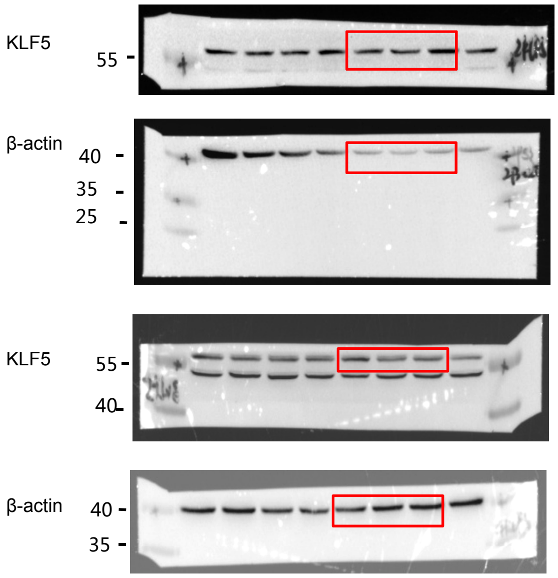


Fig. 6g

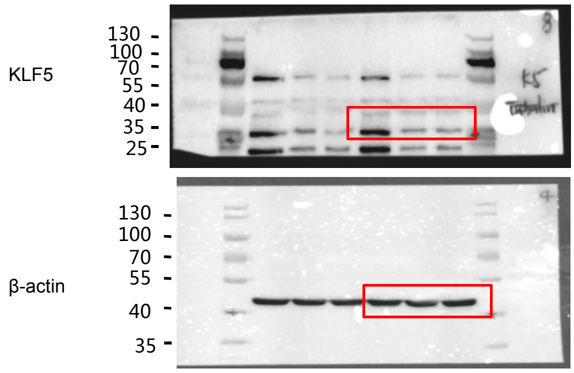

Fig. 6h

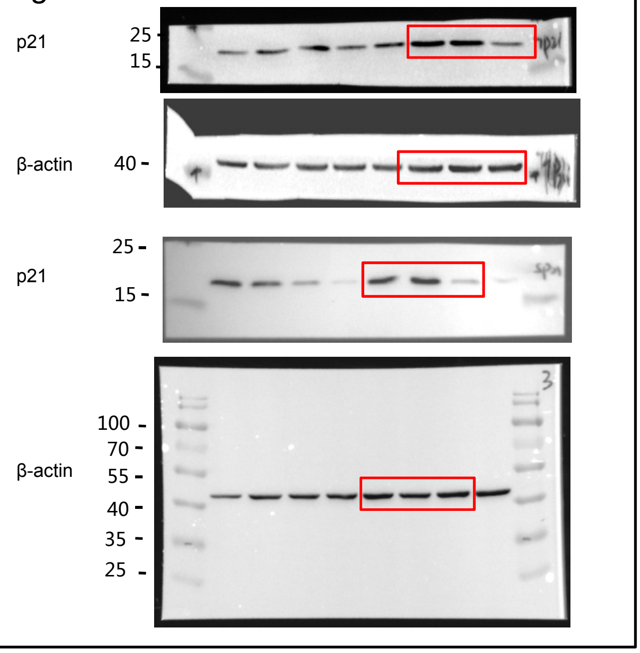


Fig. 6i

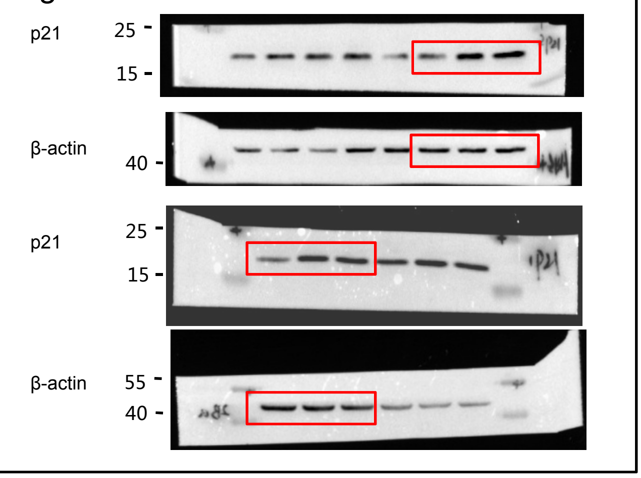


Fig. 6j

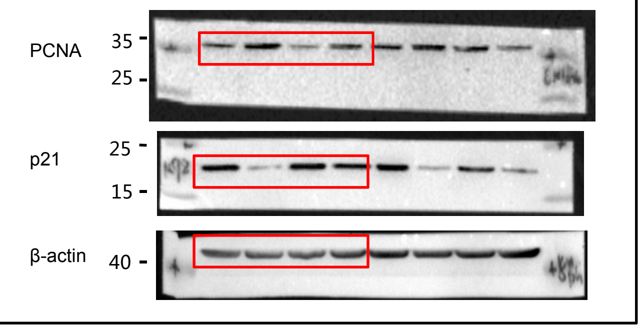


Fig. 6k

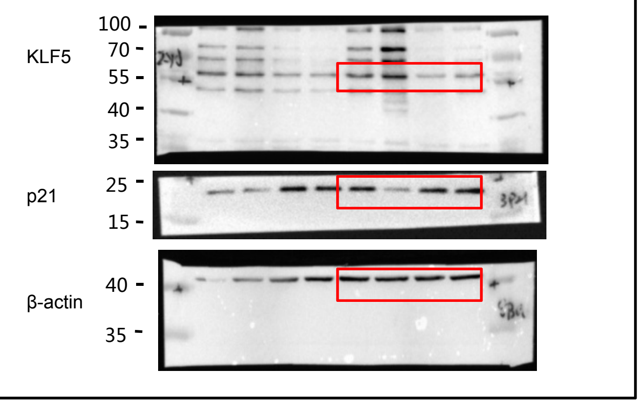


Fig. 6l

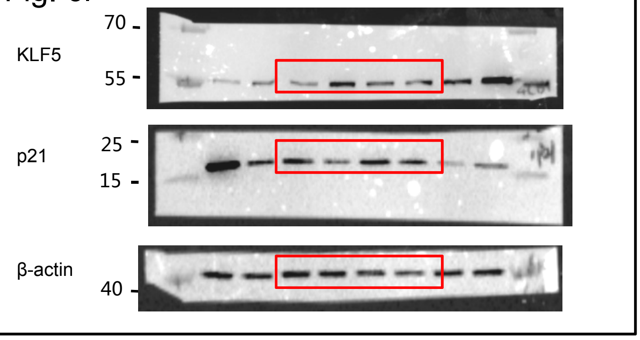


Fig. 6m

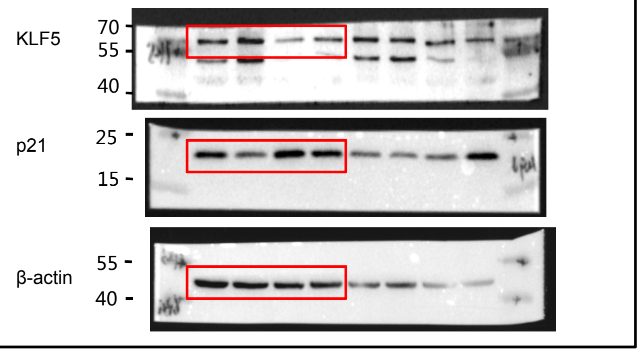


Fig. 6n

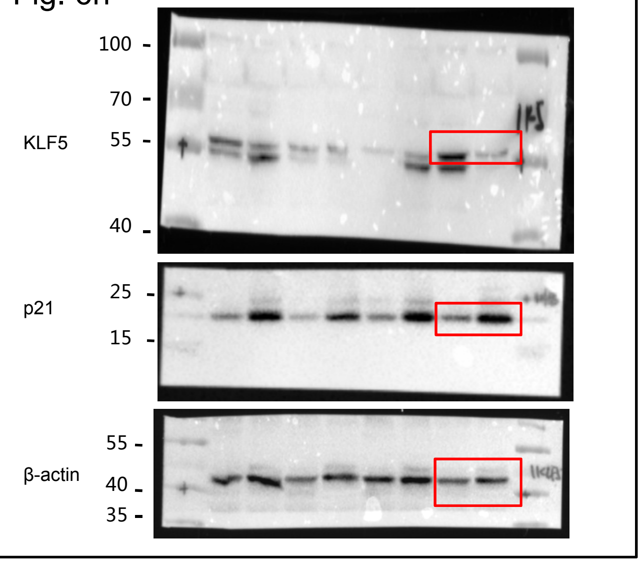


Fig. 6o

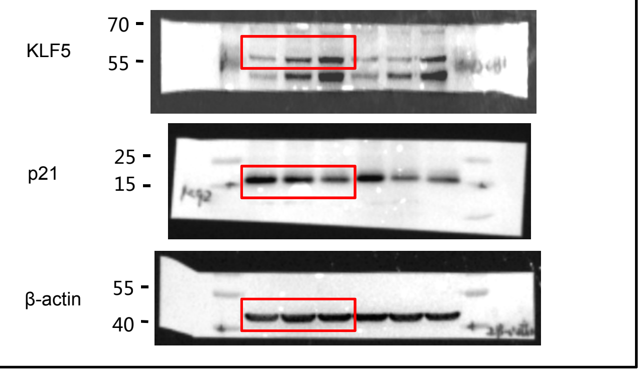

Fig. 7a

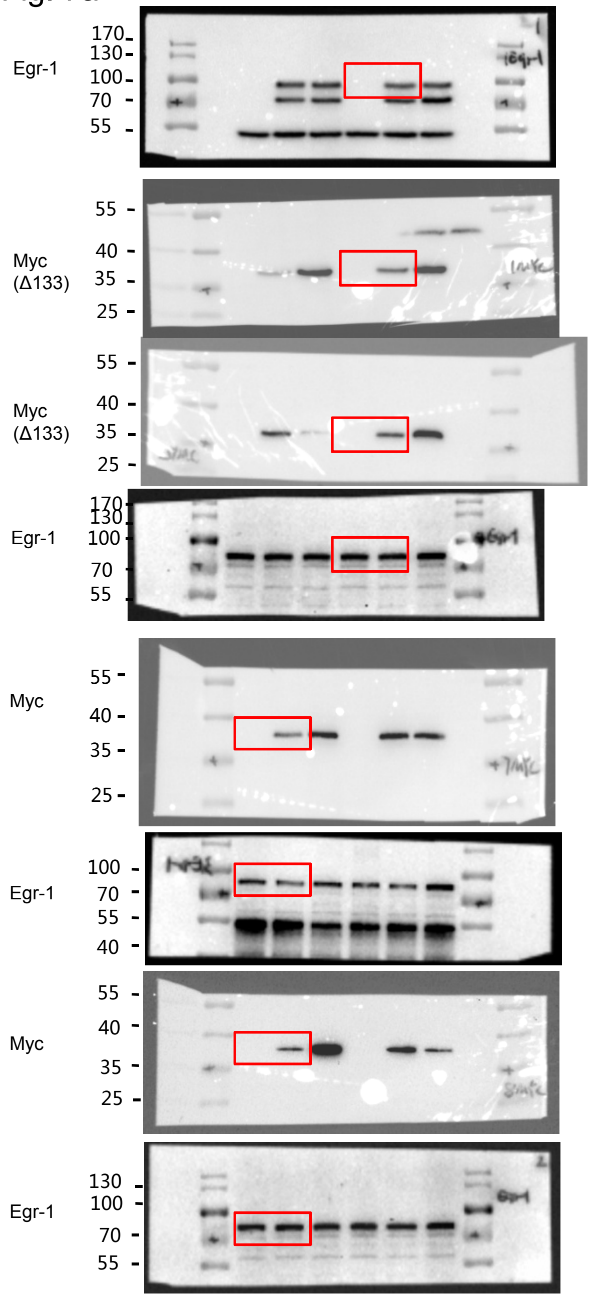


Fig. 7e

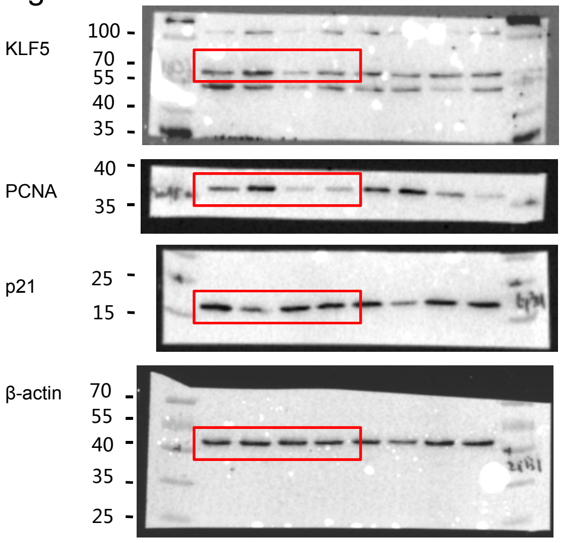


Fig. 7c

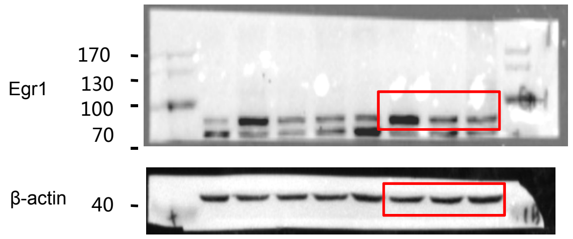


Fig. 7f

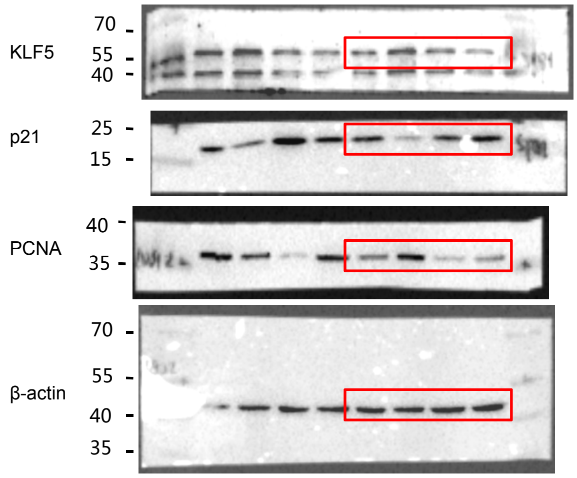


Fig. 8c

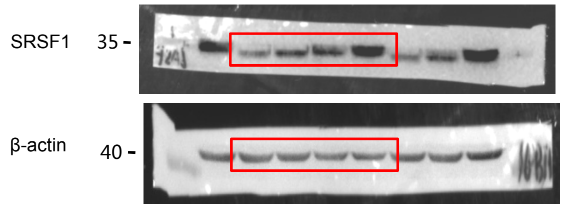


Fig. 8d

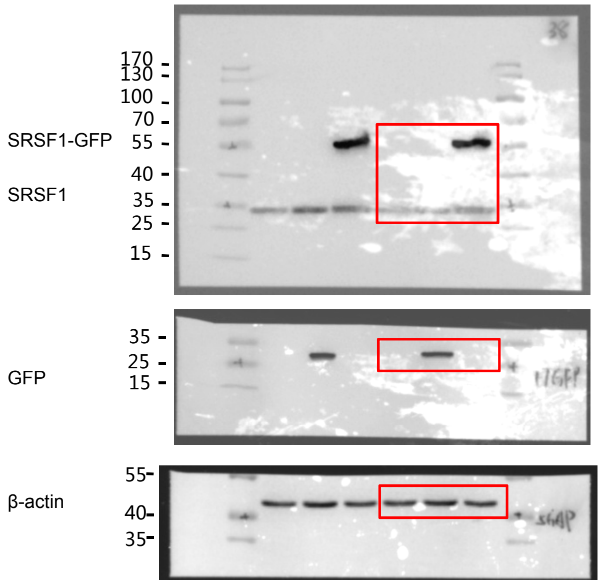

Fig. 8e

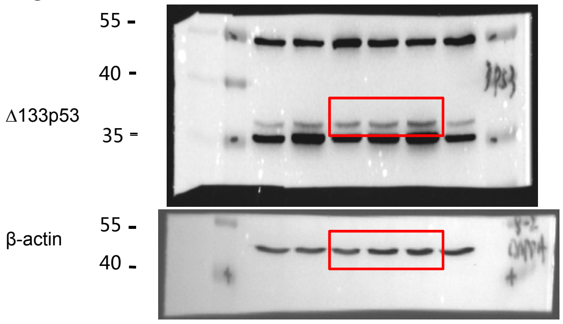


Fig. 8f

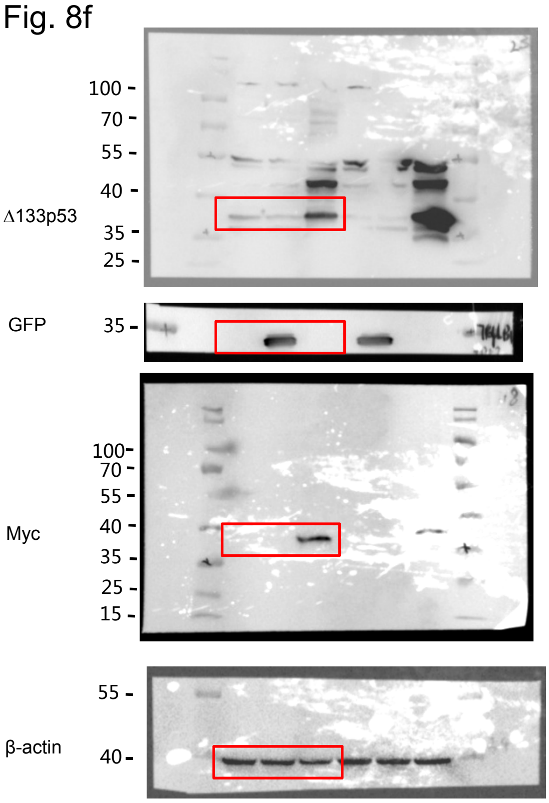


Fig. 8m

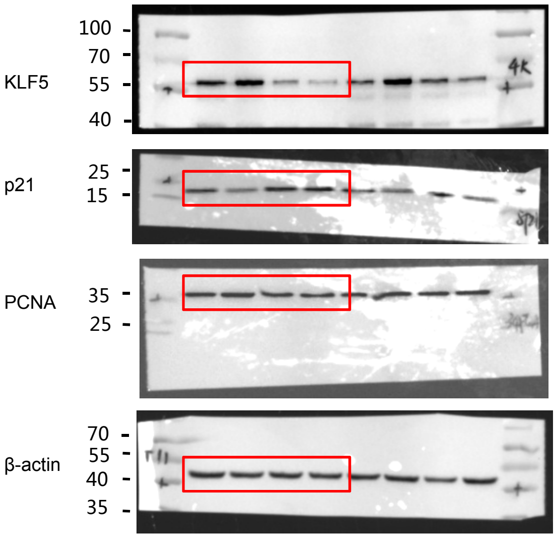


Fig. 8h

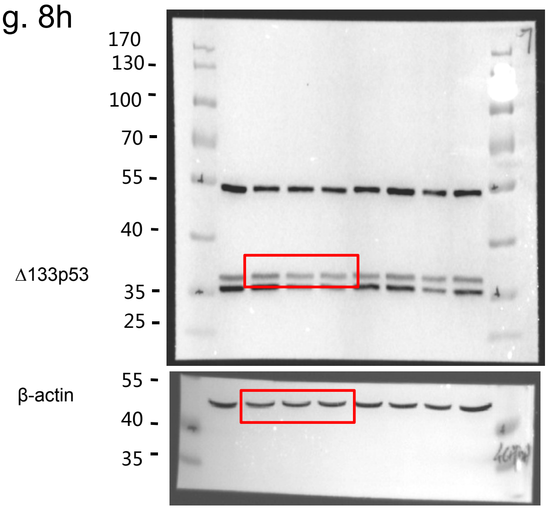


Fig. 8j

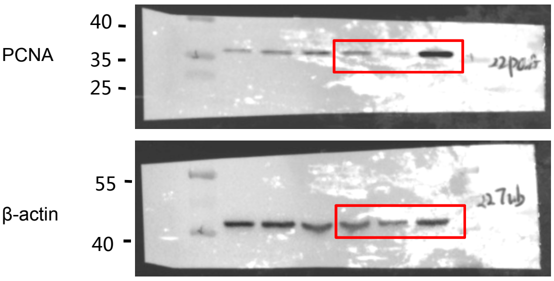


Fig. 8k

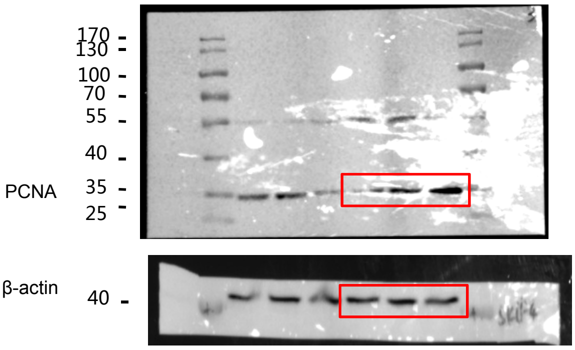


Fig. 9b

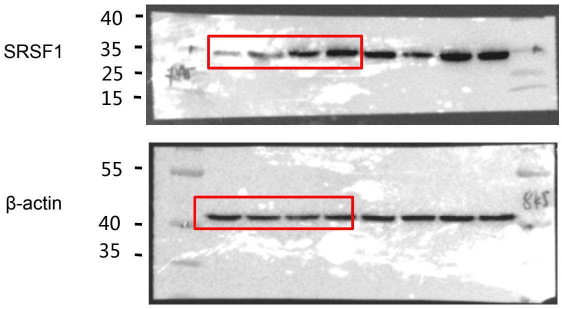

Fig. 9c

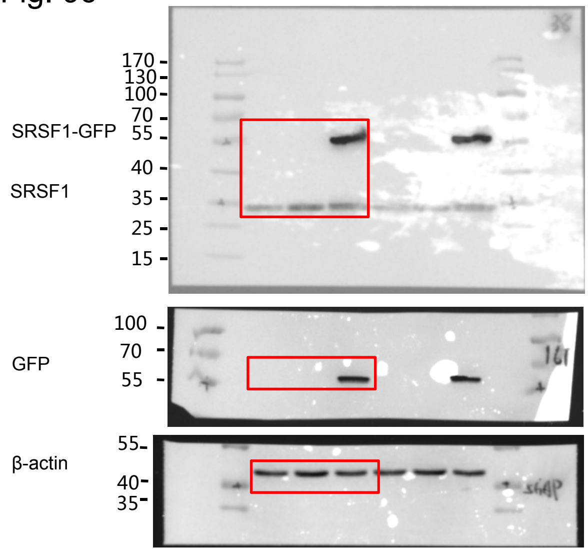


Fig. 9d

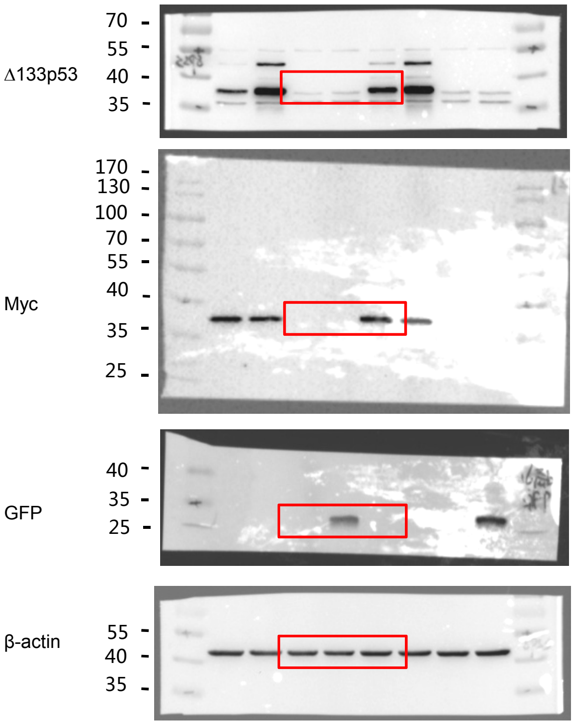


Fig. 9e

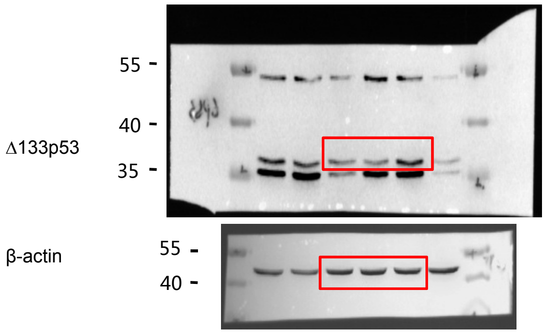


Fig. 9g

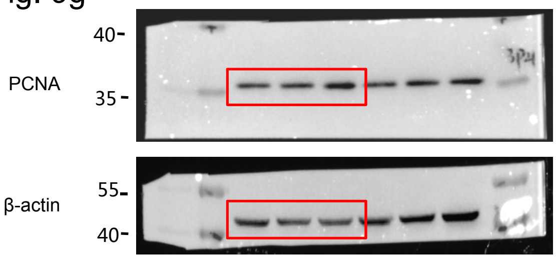


Fig. 9h

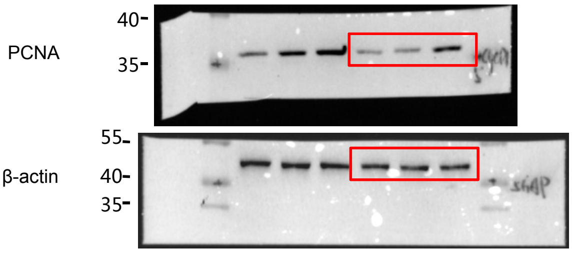


Fig. 9j

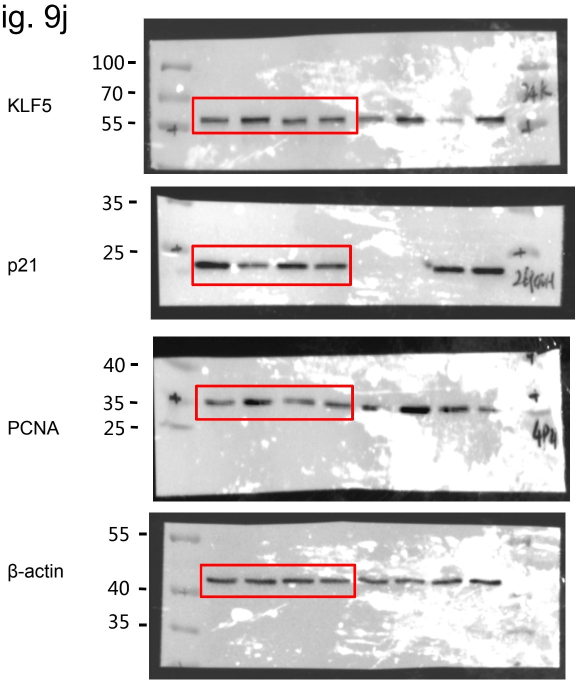


Fig. 9k

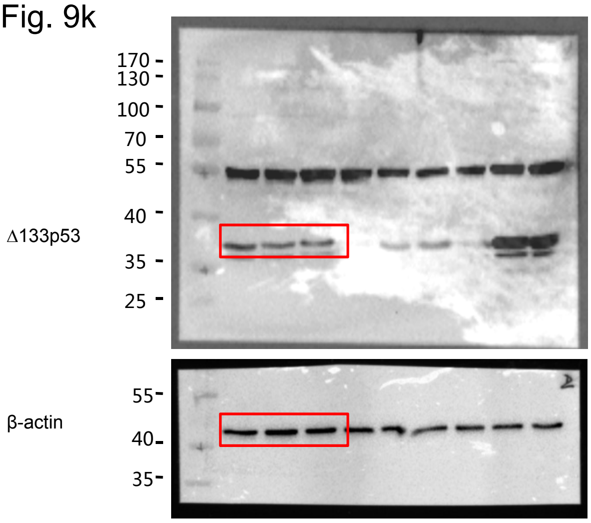

## Supplementary Fig. 1b

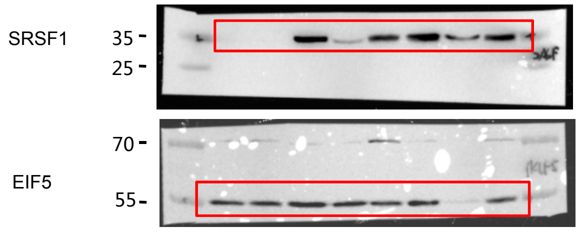


## Supplementary Fig. 1c

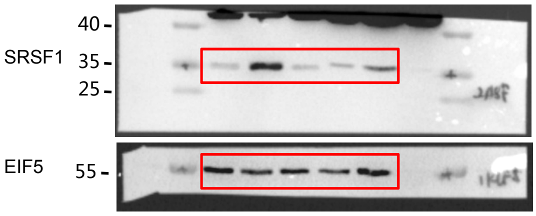


## Supplementary Fig. 7b

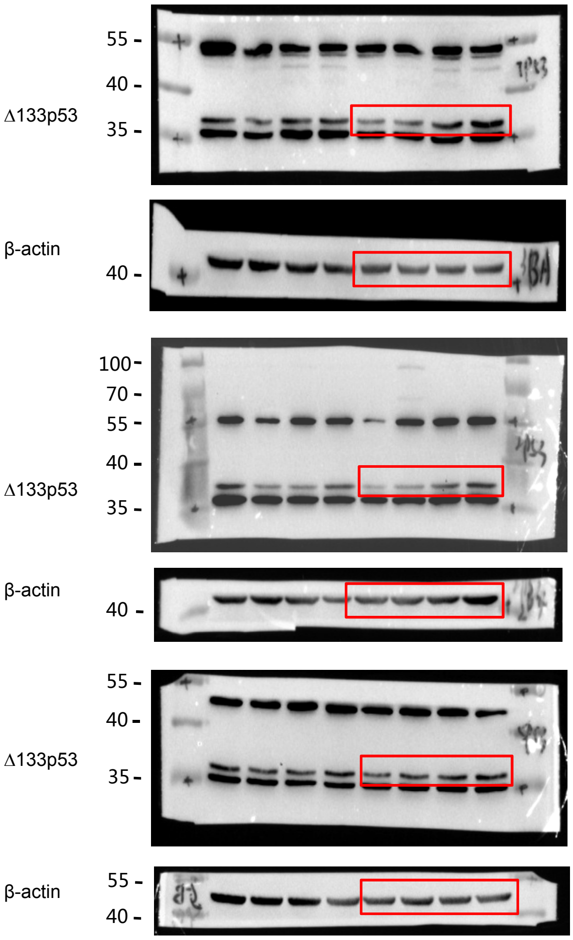


## Supplementary Fig. 2c

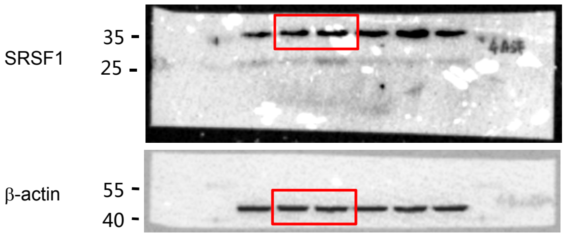


## Supplementary Fig. 3a

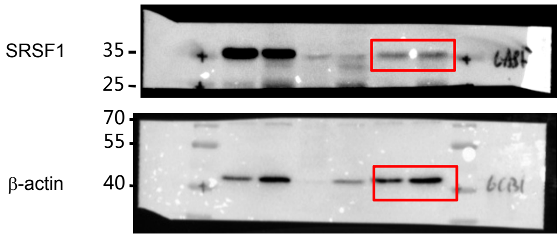


## Supplementary Fig. 5

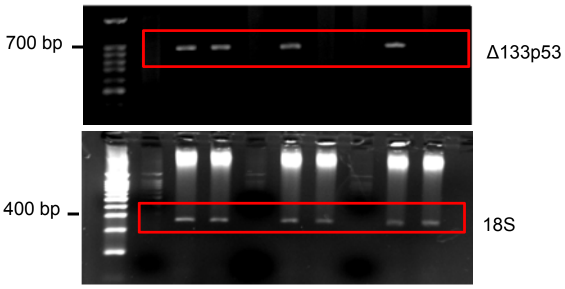


## Supplementary Fig. 8a

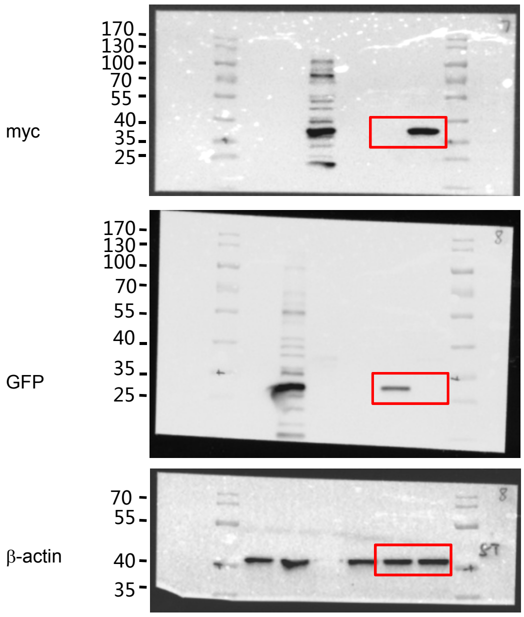

## Supplementary Fig. 8b

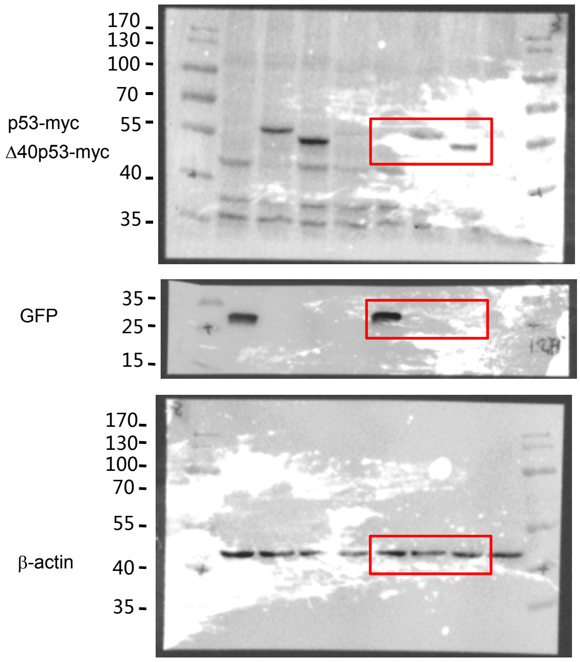


## Supplementary Fig. 8d

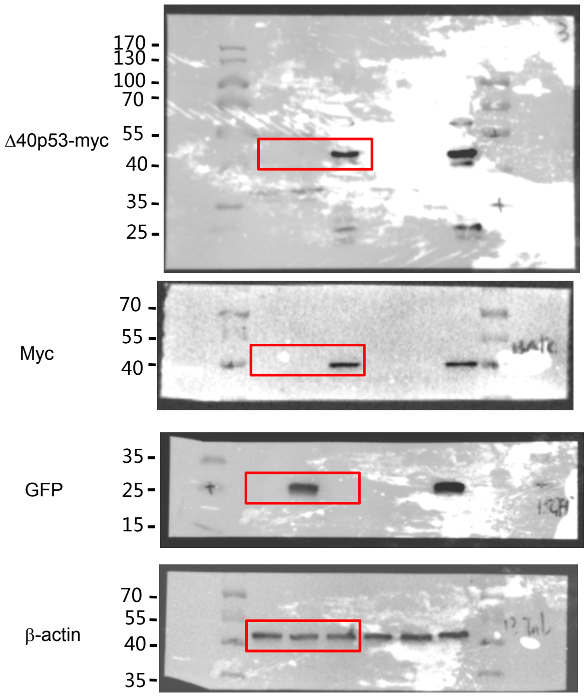


## Supplementary Fig. 8c

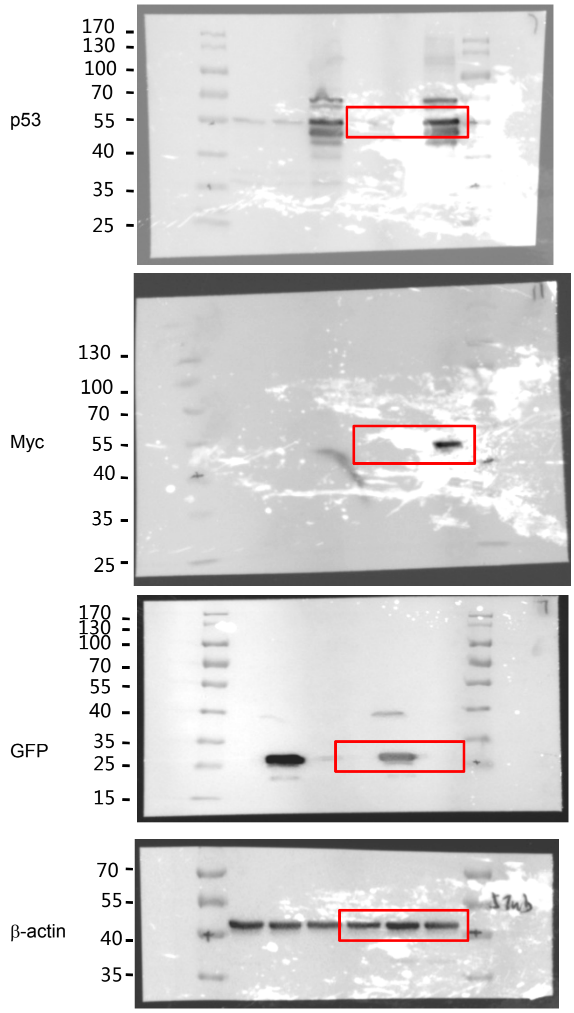


## Supplementary Fig. 9b

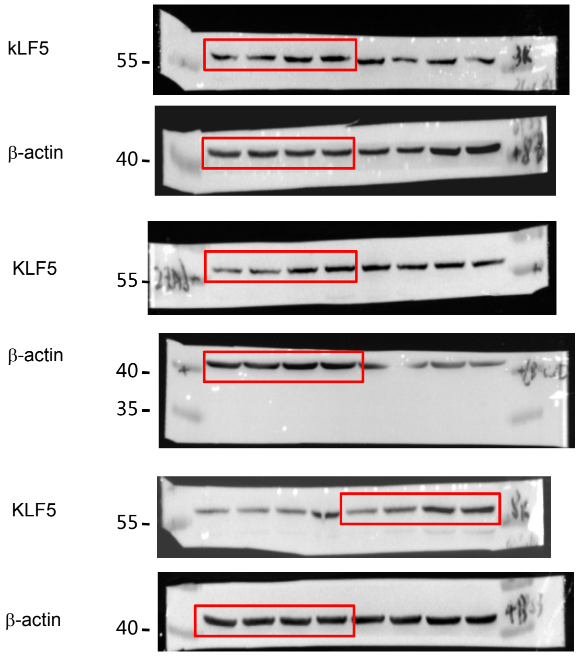


## Supplementary Fig. 10a

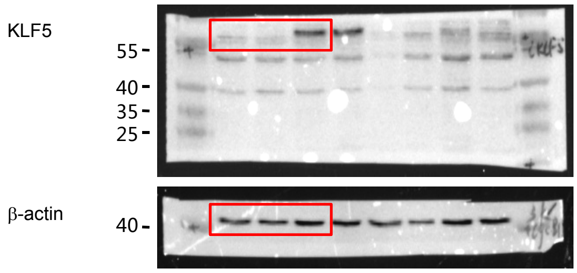

## Supplementary Fig. 10b

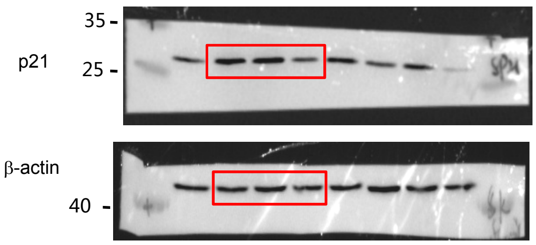


## Supplementary Fig 17c

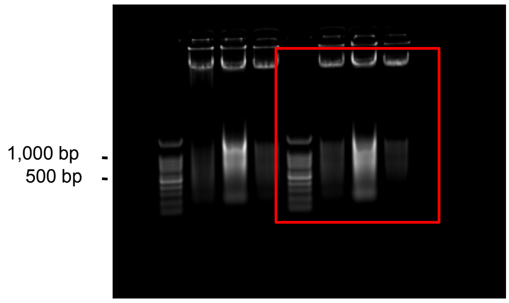


## Supplementary Fig 17e

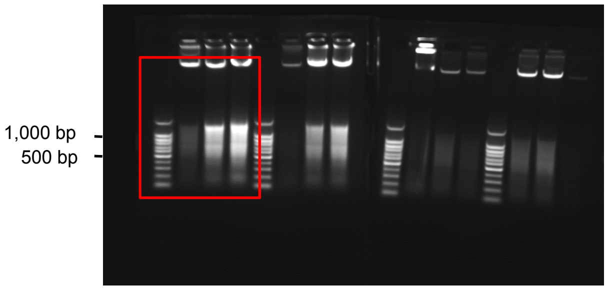


## Supplementary Fig. 17h

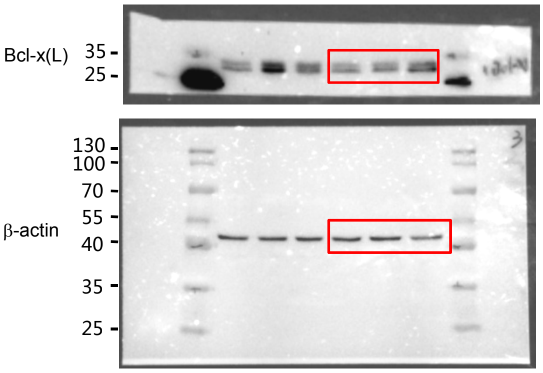


## Supplementary Fig. 17j

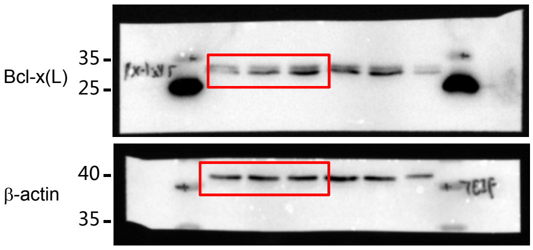


## Supplementary Fig. 15

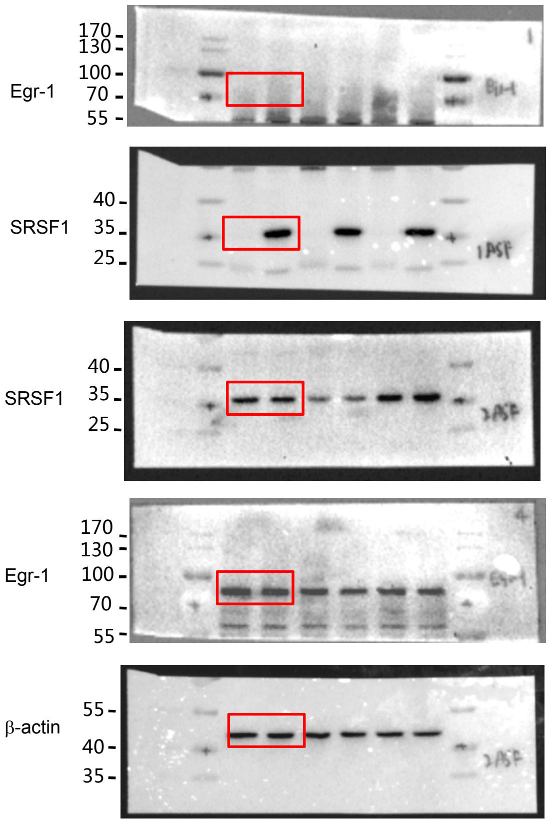


## Supplementary Fig. 17l

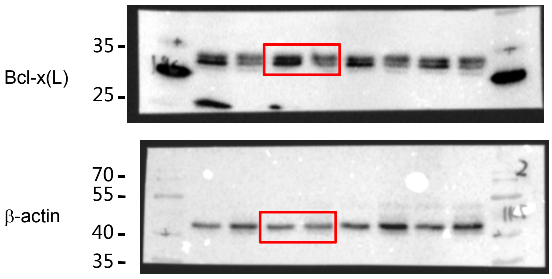


## Supplementary Fig. 17n

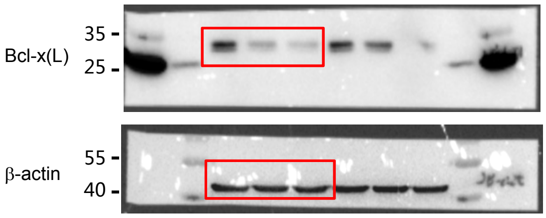


## Supplementary Fig. 17p

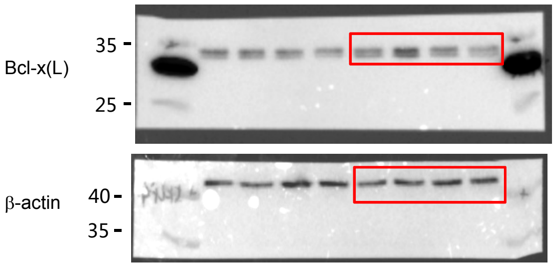

## Supplementary Fig. 17q

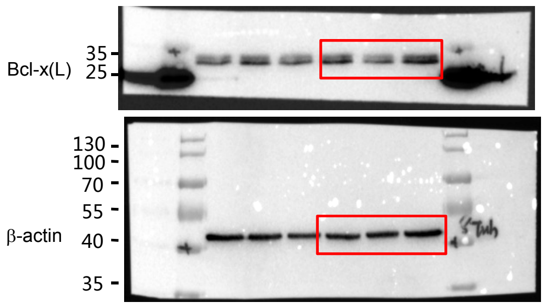


## Supplementary Fig. 19a

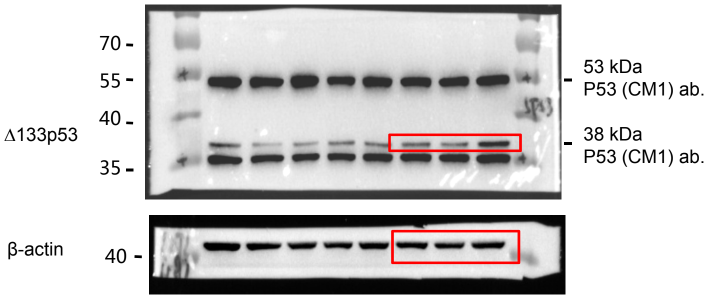


## Supplementary Fig. 18a

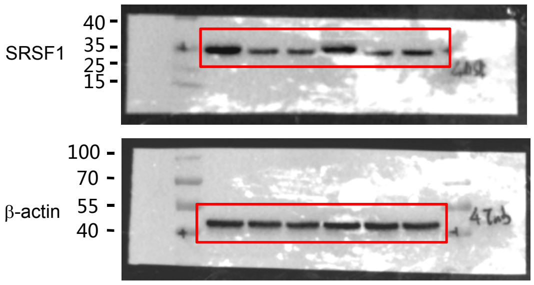


## Supplementary Fig. 18b

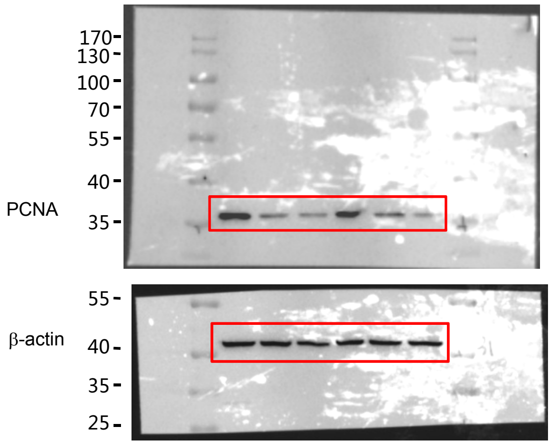


## Supplementary Fig. 19c

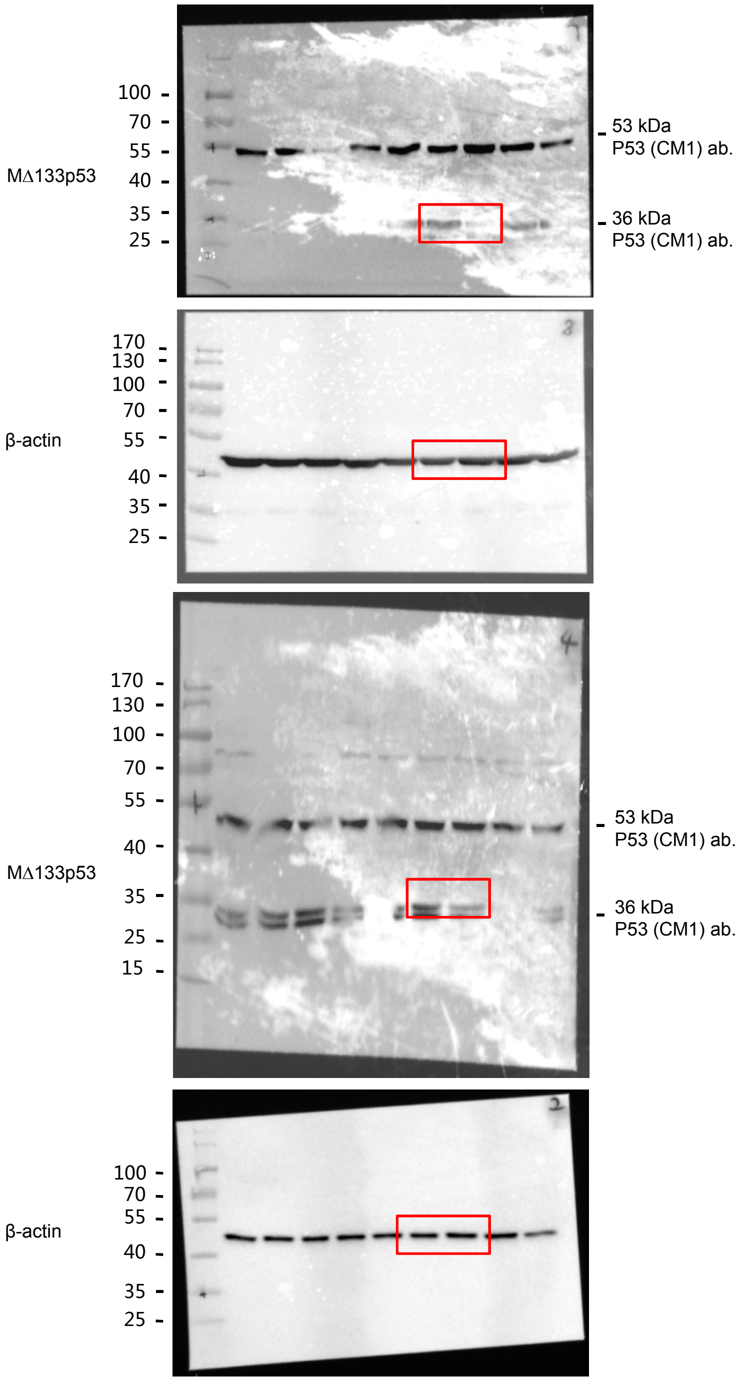

Supplementary Fig. 19b

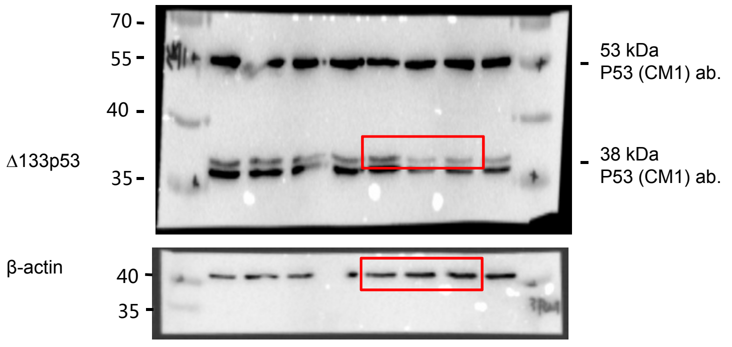


Supplementary Fig. 19d

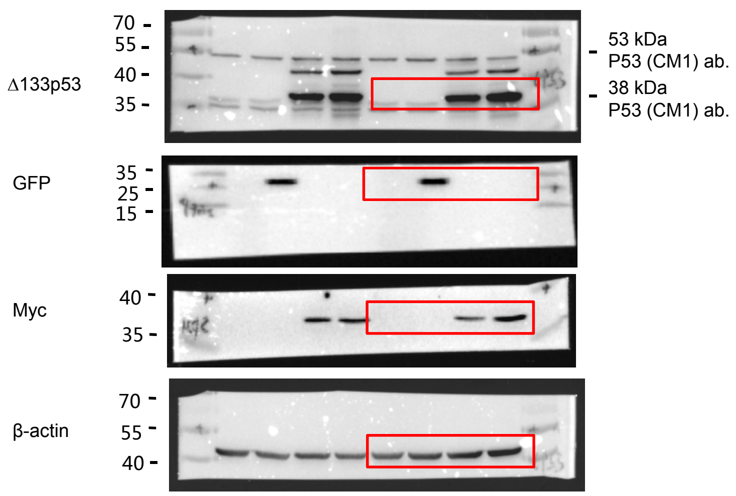


Supplementary Fig. 19e

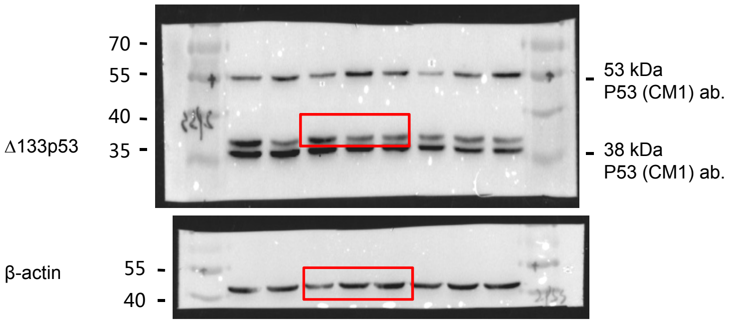

**Supplementary Table 1**

**Gene expression profiling of human aortic smooth muscle cells transfected with Ad-*Δ133p53* and Ad-*GFP*, or treated with Ang II.**

| Gene title | Gene symbol | Fold of change (Δ133p53/GFP) | Fold of change (Ang II/GFP) |
|---|---|---|---|
| Adrenomedullin 2 | ADM2 | 2.10 | 1.79 |
| Rho GTPase activating protein 26 | ARHGAP26 | 7.83 | 2.53 |
| Rho/Rac guanine nucleotide exchange factor (GEF) 2 | ARHGEF2 | 1.65 | 1.58 |
| Rho guanine nucleotide exchange factor (GEF) 28 | ARHGEF28 | 4.91 | 2.78 |
| BMP and activin membrane-bound inhibitor homolog (Xenopus laevis) | BAMBI | 3.04 | 2.46 |
| Calmodulin 2 (phosphorylase kinase, delta) | CALM2 | 2.11 | 1.61 |
| CAP, adenylate cyclase-associated protein, 2 (yeast) | CAP2 | 4.18 | 3.58 |
| Dimethylarginine dimethylaminohydrolase 1 | DDAH1 | 3.75 | 3.75 |
| Deleted in liver cancer 1 | DLC1 | 1.54 | 1.99 |
| EPH receptor A5 | EPHA5 | 1.50 | 2.57 |
| Fms-related tyrosine kinase 1 | FLT1 | 2.16 | 1.76 |
| Guanine nucleotide binding protein (G protein), beta polypeptide 4 | GNB4 | 1.87 | 1.61 |
| Histone deacetylase 9 | HDAC9 | 1.58 | 2.06 |
| Inhibin, beta A | INHBA | 8.38 | 5.40 |
| Intersectin 1 (SH3 domain protein) | ITSN1 | 1.67 | 1.59 |
| Kruppel-like factor 5 (intestinal) | KLF5 | 3.69 | 3.75 |
| Met proto-oncogene (hepatocyte growth factor recepto | MET | 2.68 | 1.59 |
| Myosin, heavy chain 10, non-muscle | MYH10 | 1.95 | 4.03 |
| Myosin, light chain 12A, regulatory, non-sarcomeric | MYL12A | 2.16 | 2.43 |
| Myosin, light chain 12B, regulatory | MYL12B | 1.53 | 1.80 |
| Myosin, light chain 9, regulatory | MYL9 | 1.65 | 1.73 |
| Neural precursor cell expressed, developmentally down-regulated 4-like, E3 ubiquitin protein ligase | NEDD4L | 1.67 | 1.58 |
| Oxytocin receptor | OXTR | 6.88 | 8.67 |
| Phosphodiesterase 1C, calmodulin-dependent 70kDa | PDE1C | 2.42 | 1.63 |
| Phosphodiesterase 3A, cGMP-inhibited | PDE3A | 1.55 | 1.58 |
| Retinol dehydrogenase 10 (all-trans) | RDH10 | 2.01 | 6.47 |
| Regulator of G-protein signaling 4 | RGS4 | 6.28 | 1.82 |
| Sodium channel, voltage-gated, type III, alpha subunit | SCN3A | 1.87 | 2.87 |
| Syndecan 2 | SDC2 | 1.63 | 1.90 |
| Sema domain, seven thrombospondin repeats (type 1 | SEMA5A | 1.45 | 3.12 |
| Serpin peptidase inhibitor, clade E (nexin, plasminogen activator inhibitor type 1), member 1 | SERPINE1 | 6.06 | 2.49 |
| SMAD specific E3 ubiquitin protein ligase 2 | SMURF2 | 1.87 | 1.53 |
| Sphingosine kinase 1 | SPHK1 | 2.52 | 2.04 |
| Transforming growth factor, beta receptor 1 | TGFBR1 | 2.18 | 1.94 |
| Thrombospondin 1 | THBS1 | 2.03 | 1.97 |
| Tribbles homolog 3 (Drosophila) | TRIB3 | 6.69 | 2.46 |
| Transient receptor potential cation channel, subfamily C, member 4 | TRPC4 | 3.30 | 3.06 |
| Transient receptor potential cation channel, subfamily C, member 6 | TRPC6 | 3.21 | 3.30 |
| Vascular endothelial growth factor C | VEGFC | 1.50 | 1.50 |

**Supplementary Table 2**

**Antibodies information**

| **Antibodies name** | **Products information** | **Working concentration** |
|---|---|---|
| SRSF1 | Invitrogen, 32-4500; recognizes the first 97 amino acids of SRSF1 | 1:1,000 for western blots |
| SRSF1 | Santa Cruz, sc-33652 | 1:200 for immunohistochemistry |
| SRSF1 | Abcam, ab129108 for Fig. 1b | 1:200 for immunofluorecence |
| SM-a-actin | Abcam, ab5694 for Fig. 1b | 1:200 for immunofluorecence |
| PCNA | Cell Signaling Technology, 2586; recognizes the protein region within the amino acid residues 111–1251 | 1:200 for immunohistochemistry, 1:1,000 for western blots |
| p21 | Cell Signaling Technology, 2947 | 1:1,000 for western blots |
| EGR1 | Cell Signaling Technology, 4153 | 1:100 for immunoprecipitation, 1:1,000 for western blots |
| GFP | Cell Signaling Technology, 2955 | 1:1,000 for western blots |
| Myc | Sigma, SAb4700447 | 1:100 for immunoprecipitation, 1:1,000 for western blots |
| p53(CM1) | Vector Laboratories, VP-P955; a rabbit polyclonal antibody raised against recombinant full-length human p53 protein that recognizes all p53 isoforms, in which Δ133p53β and Δ133p53γ are weakly recognized as these isoforms have lost most immunogenic domains of p53 | 1:1,000 for western blots |
| β-actin | Sigma, A1978 | 1:1,000 for western blots |
| KLF5 | Gene-Tex, GTX103289 | 1:1,000 for western blots |
| Bcl-xL | Cell Signaling Technology, 2762 | 1:1,000 for western blots |
| eIF-5 | Santa Cruz, sc282 | 1:1,000 for western blots |

**Supplementary Table 3**

**Primers for real-time PCR**

| **Genes** | **Sense primers (5'-3')** | **Antisense primers (5'-3')** |
|---|---|---|
| h18S | CAGCCACCCGAGATTGAGCA | TAGTAGCGACGGGCGGTGTG |
| Human SRSF1 | TCCAGACATCCGAACCAA | TACCCATCGTAATCATAGCC |
| Human KLF5 | AGCAGCAATGGACACTCTT | GCCTTCCCAGGTACACTT |
| Human p21 | GGAAGACCATGTGGACCTGT | GGCGTTTGGAGTGGTAGAAA |
| Human Δ133p53 | ACTCTGTCTCCTTCCTCTTCCTACAG | GTGTGGAATCAACCCACAGCT |
| Human TAp53 | CAGCCAAGTCTGTGACTTGCA | GTGTGGAATCAACCCACAGCT |
| Human Δ40p53 | TGAGTGGATCCATTGGAAGG | GTCTGAAAGACAAGAGCAGAAAG |
| Human Bcl-xL | CTGACAGTGAATCTGATGA | GACAAGAATGCCAAGTTAG |
| m18S | GGAAGGGCACCACCAGGAGT | GCAGCCCCGGACATCTAAG |
| Mouse SRSF1 | GTGGAAGCTGGCAGGACTTA | AGGCAGTTTCTCCCTCGTGA |
| Mouse KLF5 | GGAAGTCCCGATAGACAAGC | GCGAATTAACTGGCAGAGTG |
| Mouse Bcl-xL | CAGCGGAGAGTTAATGTT | AGGCAATGACAATCTGAG |
| Mouse p21 | TGGGCATGTCAGAAGTCTCG | CTCGCAGCTTTCCAATGCAC |
| r18S | GGAAGGGCACCACCAGGAGT | TGCAGCCCCGGACATCTAAG |
| Rat SRSF1 | CTCCGAGTGGAAGCTGGCAGGACT | CACCAGTGCCATCTCGGTAAACA |
| Rat KLF5 | TTGAAGCTGCAGTATGCCTG | CTGAGGCACTGTCTCGTCTG |
| Rat Bcl-xL | CAGTGGAGAGCTAATGTT | AGGCGATGACAATCTGAG |
| Rat p21 | CAAAGTATGCCGTCGTCTGTTC | TCAAAGTTCCACCGTTCTCG |

## Supplementary Table 4

## Sequences of siRNAs against SRSF1, Δ133p53, KLF5, and EGR1

| | | | |
|---|---|---|---|
| Human SRSF1 | siRNA1 | Sense (5'-3') | GCAGAUGCAGUAGGCGUAA |
| | | Antisense (5'-3') | UUACGCCUACUGCAUCUGC |
| | siRNA2 | Sense (5'-3') | GCAGAGAGCAGUAAUCCAA |
| | | Antisense (5'-3') | UUGGAUUACUGCUCUCUGC |
| Human Δ133p53 | siRNA1 | Sense (5'-3') | UGUUCACUUGUGCCCUGACUUUCAA |
| | | Antisense (5'-3') | UUGAAAGUCAGGGCACAAGUGAACA |
| | siRNA2 | Sense (5'-3') | CUUGUGCCCUGACUUUCAATT |
| | | Antisense (5'-3') | UUGAAAGUCAGGGCACAAGTT |
| Human KLF5 | siRNA1 | Sense (5'-3') | GCAGACUGCAGUGAAACAA |
| | | Antisense (5'-3') | UUGUUUCACUGCAGUCUGC |
| | siRNA2 | Sense (5'-3') | GCAUCCACUACUGCGAUUA |
| | | Antisense (5'-3') | UAAUCGCAGUAGUGGAUGC |
| Human EGR1 | siRNA1 | Sense (5'-3') | CCAACAGUGGCAACACCUU |
| | | Antisense (5'-3') | AAGGUGUUGCCACUGUUGG |
| | siRNA2 | Sense (5'-3') | GCCUAGUGAGCAUGACCAA |
| | | Antisense (5'-3') | GCCUAGUGAGCAUGACCAA |
| Human PCNA | siRNA1 | Sense (5'-3') | ACGCGGATACCTTGGCGCTAGTATT |
| | | Antisense (5'-3') | UUTUCTUGCGCCUUGGTUTCCGCGT |
| | siRNA2 | Sense (5'-3') | CCAGGAGAAAGTTTCAGACTATGAA |
| | | Antisense (5'-3') | TTCUTUGTGTGUUUCTTTCTCCTGG |

**Supplementary Table 5**

**ChIP-PCR primers for EGR1 binding peaks**

| **Genes** | **Sense primers (5'-3')** | **Antisense primers (5'-3')** |
|---|---|---|
| P1 (nt-2722 to nt -2577) | CCACTGCCTCCTTCAAAC | CTTAGTCACTGGGCAACAC |
| P2 (nt-105 to nt -10) | TCTCCACCCTAATTCCC | GTTTGTACACAACTTCTCTG |
| P3 (nt-410 to nt -558) | GAGGAGTCCACCCGAAA | TGCTCAGCACCCTTGTA |
| P4 (nt-1475 to nt -1576) | GCAGCCGAATTAGAGCAA | TCCTTGGGACAGGCTAAA |

**Supplementary Table 6**

**Primers for amplification of human Δ133p53 isoforms by RT-PCR (nested PCR)**

| Primers name | 5'-3' sequence |
|---|---|
| N1Δ133(F9) | TAGACGCCAACTCTCTCTAG |
| N1Δ133(R9) | GACGCACACCTATTGCAAGCAAGGGTTC |
| N2Δ133(F10) | CTAGTGGGTTGCAGGAGGTGCTTACAC |
| N2Δ133(R10) | ATGTCAGTCTGAGTCAGGCCCTTCTGTC |
| N2Δ133b(F11) | CTAGTGGGTTGCAGGAGGTGCTTACAC |
| N2Δ133b(R11) | TTTGAAAGCTGGTCTGGTCCTGA |
| N2Δ133g(F12) | CTAGTGGGTTGCAGGAGGTGCTTACAC |
| N2Δ133g(R12) | TCGTAAGTCAAGTAGCATCTGAAGG |

**Supplementary Table 7**

**Primers for quantitative amplification of human p53 isoforms by RT-qPCR**

| **Primers name** | **5'-3' sequence** |
|---|---|
| Δ133 (I4) F3 | ACTCTGTCTCCTTCCTCTTCCTACAG |
| Δ133 (E5) R3 | GTGTGGAATCAACCCACAGCT |
| p53alpha (E9) F4 | AACCACTGGATGGAGAATATTTCAC |
| p53alpha (E10) R4 | CAGCTCTCGGAACATCTCGAA |
| p53beta (E9) F5 | AACCACTGGATGGAGAATATTTCAC |
| p53beta (E9b) R5 | TCATAGAACCATTTTCATGCTCTCTT |
| p53gamma (E9) F6 | AACCACTGGATGGAGAATATTTCAC |
| p53gamma (E9g) R6 | TCAACTTACGACGAGTTTATCAGGAA |
